# Single-cell analysis reveals heterogeneity of juvenile idiopathic arthritis fibroblast-like synoviocytes with implications for disease subtype

**DOI:** 10.1186/s13075-022-02913-8

**Published:** 2022-09-27

**Authors:** Megan M. Simonds, Kathleen E. Sullivan, AnneMarie C. Brescia

**Affiliations:** 1 Nemours Biomedical Research, Nemours Children’s Health, Delaware, 1701 Rockland Rd, Wilmington, DE 19803 USA; 2 Allergy and Immunology, Children’s Hospital of Philadelphia, Philadelphia, PA USA; 3 Division of Rheumatology, Nemours Children’s Health, Wilmington, DE USA

**Keywords:** Juvenile idiopathic arthritis, Fibroblast-like synoviocytes, Chondrocytes, Genomics

## Abstract

**Background:**

Fibroblast-like synoviocytes (FLS) play a crucial role in JIA pathogenesis; however, the mechanisms by which they contribute to disease progression are not well described. Previous studies demonstrated that rheumatoid arthritis FLS are heterogeneous, and subpopulations with transformed, aggressive phenotypes cause invasive and destructive disease activity. We employ single-cell RNA-sequencing (scRNA-seq) to investigate JIA FLS heterogeneity and gene expression that distinguishes JIA subtypes.

**Methods:**

JIA FLS cell lines from three persistent oligoarticular, three pre-extension oligoarticular, and three polyarticular subtypes were cultured. scRNA-seq was performed by Genewiz according to 10 × Genomics Chromium protocols. SeuratR package was used for QC, analysis, and exploration of data.

**Results:**

FLS are heterogeneous and have characteristics of fibroblasts, chondrocytes, and smooth muscle cells. The chondrocyte-like subpopulation is the predominant cell type and percentages of this subpopulation increase with disease severity. Despite overlapping subpopulations, the chondrocyte-like cells have unique genetic fingerprints that distinguish between JIA subtypes. LRRC15, GREM1, and GREM2 are overexpressed in chondrocyte-like cells from persistent oligoarticular JIA FLS compared to pre-extension oligoarticular JIA FLS. S100A4, TIMP3, and NBL1 are overexpressed in pre-extension oligoarticular JIA FLS compared to polyarticular JIA FLS. CRLF1, MFAP5, and TNXB are overexpressed in persistent oligoarticular JIA FLS compared to polyarticular JIA FLS.

**Conclusions:**

We found biologically relevant differences in gene expression between JIA subtypes that support a critical role for FLS in pathogenesis. We also demonstrate that gene expression within the chondrocyte-like subpopulation can be used to distinguish between these subtypes.

**Supplementary Information:**

The online version contains supplementary material available at 10.1186/s13075-022-02913-8.

## Background

Juvenile idiopathic arthritis (JIA) is the most common rheumatic disease of childhood and carries a risk of permanent joint damage and disability. The current classification scheme for JIA includes seven subtypes of disease based on onset [1, 2]. The oligoarticular subtype is the most benign, yet disability results when joint involvement evolves from an oligoarticular to a polyarticular course, termed extended oligoarticular disease. Persistent oligoarthritis (oligo) affects up to 4 joints throughout the disease course, whereas extended oligoarthritis affects a cumulative total of 5 or more joints after the first 6 months of disease [2]. Between 21 and 50% of patients with oligoarticular onset show extension to a polyarticular (poly) course, of whom only 13–23% achieve remission [3–8]. Compared to patients with persistent oligoarticular course or polyarticular onset, patients with extended oligoarthritis have the lowest physical and mental scores in health-related quality-of-life assessments [9], with 38% requiring joint replacement, compared with only 13% of those with a persistent oligoarticular course [10].

While the pathogenesis of JIA is not completely understood, fibroblast-like synoviocytes (FLS) are thought to play a critical role in JIA progression and may contribute to joint growth disturbances in more severe forms of the disease [11, 12]. The mechanisms underlying disease-related changes in the FLS population are not clear. FLS are pluripotent cells derived from mesenchymal stem cells [13]. We reported previously that JIA FLS have a chondrocyte-like phenotype based on mRNA and protein expression of cartilaginous markers including collagen II, cartilage oligomeric matrix protein, aggrecan, and collagen X [11]. Human synovial cells produce COMP and glycosaminoglycans similar to chondrocytes [14, 15], and a fibroblast-like cell with features of both fibroblasts and chondrocytes has been described in the pannus of RA [16]. Based on previous work in RA, we show that FLS evolve toward a chondrocyte-like phenotype.

Previous work has used bulk RNA-seq to identify changes in FLS associated with disease. This strategy does not identify heterogeneity in cells, and recent evidence in RA has demonstrated that subtypes of FLS contribute disproportionately to disease progression. We therefore hypothesized that JIA subtypes might be associated with distinct cell subpopulations. Single-cell RNA-sequencing (scRNA-seq) is a technology for high-throughput sequencing and analysis of genes and transcripts at the single-cell level. In this study, this technology was used to identify subpopulations within JIA FLS and show differences between subtypes of JIA and between cell subpopulations within the subtypes. We know that there is heterogeneity among FLS in RA that can be detected by single-cell RNA-sequencing and that this heterogeneity is related to disease activity [12, 17–24]. We have used scRNA-seq to analyze the transcriptome of JIA FLS at the single-cell level. Cell-to-cell variability is not adequately assessed using arrays or bulk RNA-sequencing. Identifying heterogeneity in the FLS population prior to extension is central to not only understanding the role of these cells in extension, but also to the overall pathogenesis of JIA. Our intriguing scRNA-seq data demonstrating multiple subpopulations of FLS led to our conclusion that using scRNA-seq, genome and transcriptome differences can differentiate between JIA subtypes and between the cell subpopulations within subtypes. This data can be used to understand the complex pathology of this disease.

## Methods

### Selection of samples

Synovial fluid and synovial tissue samples were obtained from our Institutional Review Board-approved repository. Patients who underwent clinically indicated arthrocentesis were offered inclusion into the repository, and informed consent was obtained. We selected primary cells from three subjects with persistent oligoarticular JIA; three subjects prior to disease extension, termed extended-to-be (ETB); and three subjects with polyarticular JIA. All subjects had no prior steroid injections and were not on medications or only on nonsteroidal anti-inflammatory drugs. All subjects had no additional pathology or diagnoses that could influence results.

### Cell culture

Synovial fluid from the 9 subjects was centrifuged at 300 rpm for 5 min. Fluid was aspirated and cell pellets were cultured in an FLS growth medium (415–500 Cell Applications, Inc.) When cells reached confluence at passage 3, media were aspirated, and cells were treated with trypsin and counted. Once cell number was determined, cells were spun again at 300 rpm for 5 min and resuspended in freezing media containing 10% DMSO, diluted to 2 × 10^6^ cells, and stored at − 80 in a cell chiller for 4 h. Cells were then stored in liquid nitrogen until shipment to GENEWIZ.

### Single-cell RNA-sequencing

Once cells were confluent, they were frozen and shipped according to protocols provided by the company GENEWIZ, a division of Azenta Life Sciences, who then performed scRNA-sequencing using 10 × Genomics Chromium according to manufacturer protocols. Briefly, cells and barcoded gel beads were isolated in partitioning oil droplets using 10 × Genomics Chromium. Reverse transcription incorporated cell and transcript-specific barcodes. Barcoded libraries were pooled and sequenced using the Illumina platform [25].

### CellRanger aggregate

CellRanger aggregate was performed to combine single datasets from each biological sample. Briefly, multiple samples from a single JIA subtype were processed through multiple GEM wells and multiple libraries were generated. Those libraries were pooled onto one flow cell. After demultiplexing, CellRanger count software was run separately for each GEM well. Data was then aggregated using CellRanger aggregate software [26].

### Loupe cell browser

Using CLOUPE files generated by CellRanger aggregate, each JIA subtype was analyzed for fibroblast marker expression. The gene or feature expression imports log2 expression levels of each gene of interest and provides cell number. Cell percentages were calculated based on the total cell number.

### Seurat analysis

Seurat analysis was performed. Each group of replicates was used as separate inputs in the Seurat data analysis package. The samples were integrated using the Seurat data analysis package. Preprocessing of the data includes total expression normalization and removing unwanted sources of variation. Seurat applies a graph-based clustering approach to partition the cells into “communities.” When Seurat performs tSNE dimensionality reduction, the graph-based clusters are expected to co-localize in the projection. For each cluster in each sample, positive differentially expressed genes are calculated and their relative expression levels are visualized on a tSNE projection. The “roc” test is performed, and the resulting genes are sorted by AUC. Cells were automatically annotated using the SingleR data package from Bioconductor. Given a reference dataset of cells with known labels (in this case Human Primary Cell Atlas Data), it labels new cells from a test dataset based on similarity to the reference set. Seurat is then used to integrate the sample datasets based on common sources of variation, enabling the identification of shared populations across data sets and downstream comparative analysis. Positive differentially expressed genes are also calculated for each cluster between conditions. This allows the identification of differentially expressed markers across conditions. Seurat provided cell percentages used for each comparison.

## Results

### Despite the heterogeneity of FLS, most total cells still retain fibroblast characteristics

We established the integrity of the total cell population. FLS are pluripotent cells. To strengthen the evidence that these cells are all fibroblasts at initial input before analysis and have true heterogeneity between subtypes and subpopulations of cells, we overlayed universal markers using Loupe Cell Browser and calculated the percentages of total cells that express a group of fibroblast markers. Muhl et al. established a group of markers based on literature as no single transcript serves as a universal fibroblast marker [27]. As a group, these markers can identify fibroblasts within total cell populations (LUM, LOXL1, COL1A1, COL5A1, PDFGRA, COL1A2, FBLN1, FBLN2, and CD34). Transcripts chosen from several markers are needed to robustly distinguish fibroblasts from other subpopulations.

**Fig. 1:**
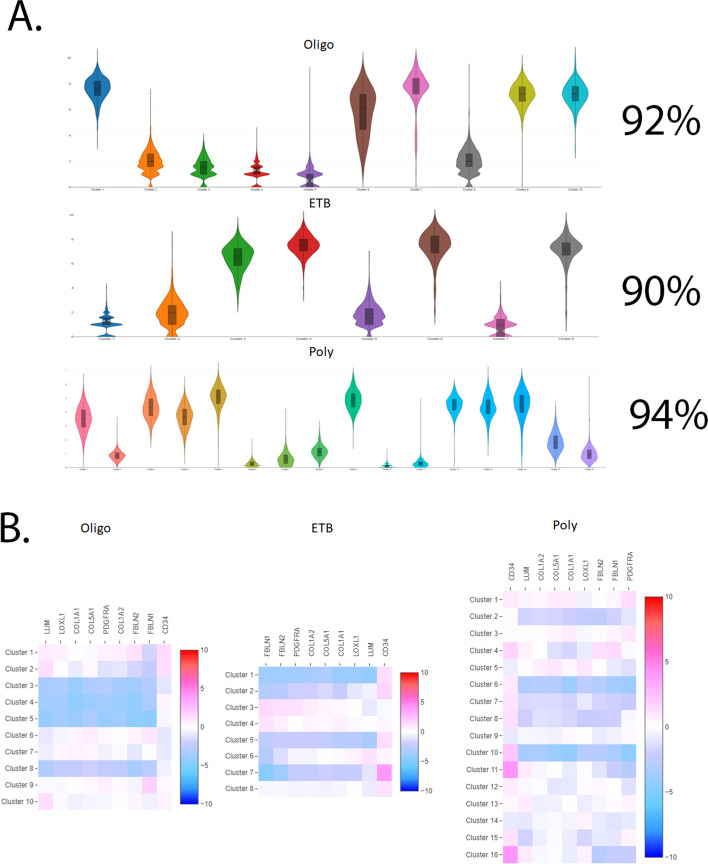
Expression of universal fibroblast markers. Markers consist of FBLN1, FBLN2, PDGFRA, COL1A2, COL5A1, COL1A1, LOXL1, LUM, and CD34. Determined that 92% of oligoarticular JIA FLS express fibroblast markers, 90% of ETB JIA FLS express fibroblast markers, and 94% of poly JIA FLS express fibroblast markers (**A**), establishing that our initial total cell input has fibroblast origins. Heatmaps provide visualizations of the expression of the individual fibroblast markers and their expression pattern in individual cell clusters (**B**). A combination of upregulated and downregulated expression of these markers in individual clusters defines cells as fibroblast

We determined that 92% of oligoarticular (oligo) JIA FLS express fibroblast markers, 90% of prior to extension (ETB) JIA FLS express fibroblast markers, and 94% of poly JIA FLS express fibroblast markers (Fig. 1A). Heatmaps provide visualizations of the expression of the individual fibroblast markers and their expression pattern in individual cell clusters (Fig. 1B). It is important to note that FLS are identified by both upregulation and downregulation of these specific genes and we confirm that no single marker can be used to identify a FLS but a combination of these markers can distinguish this cell type (Fig. 1B). This data confirms that total JIA FLS are indeed fibroblast-like synoviocytes. All cell clusters had the expression of fibroblast markers. While we establish that our initial total cell input has fibroblast origins, the following scRNA-seq analysis will reveal that these cells are heterogeneous when we compare subtypes of JIA. Additionally, within each subtype, there are conserved subpopulations of cells, and those subpopulations provide another layer of heterogeneity to the genetic fingerprint of JIA FLS.

### Despite a single cell type used for initial input for all JIA subtypes, there is heterogeneity between the subtypes of disease

**Fig. 2:**
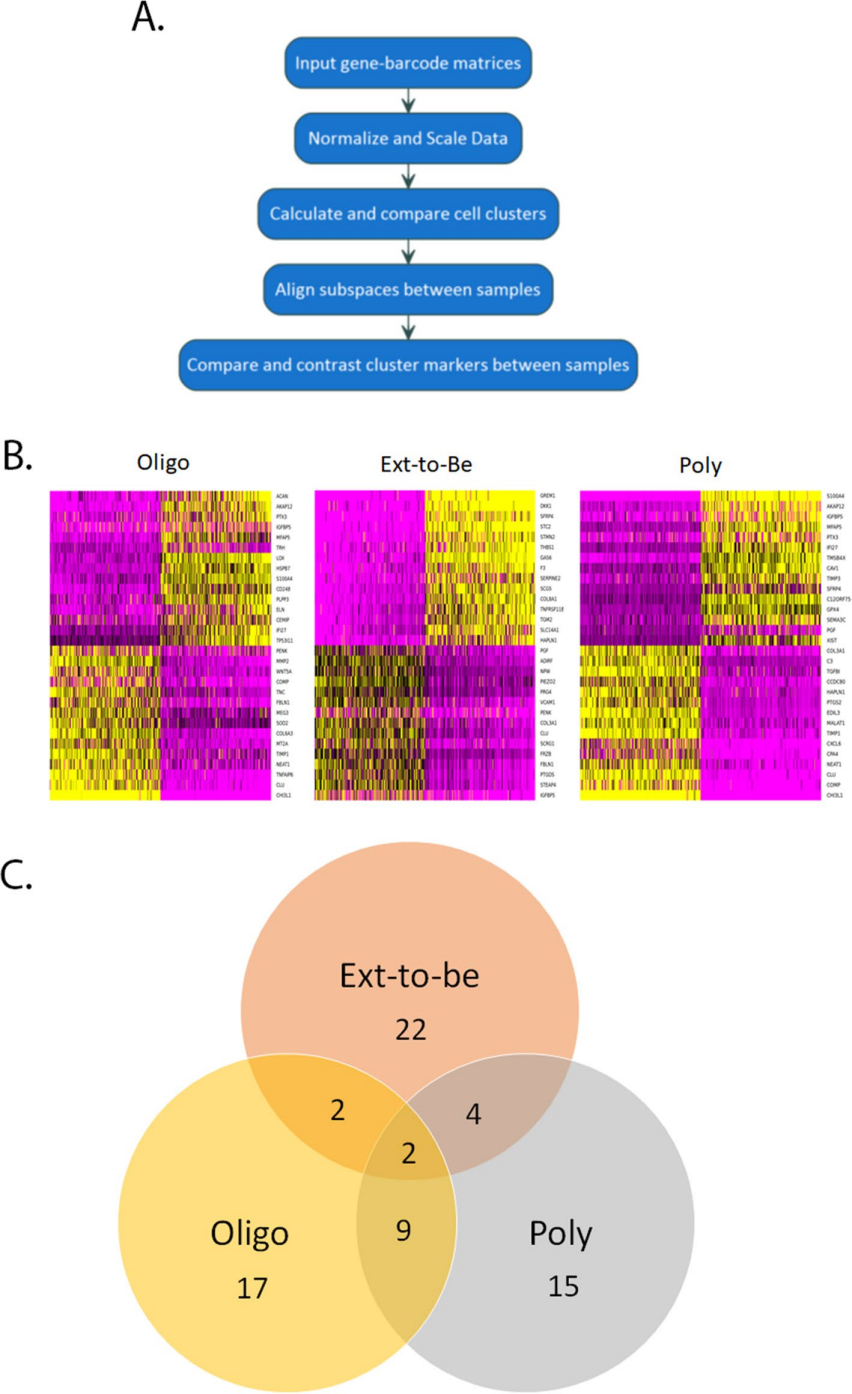
FLS are heterogeneous. Workflow diagram for initial data input and processing to provide a summary of how data was processed (**A**). Heatmaps reveal the 30 top variable genes that have the greatest differential expression within single cells (**B**). Of the 30 genes, there were 16 unique differentially expressed genes within cells from oligo, 22 unique differentially expressed genes within cells from ETB, and 15 unique differentially expressed genes within cells from poly (**C**). There were 4 genes that overlapped between oligo and ETB, 11 genes that overlapped between oligo and poly, and 6 genes that overlapped between ETB and poly (**C**). These genes can be used to differentiate between JIA subtypes

CellRanger aggregate was performed on a single JIA subtype to aggregate datasets into a single output for multi-sample analysis. Figure 2A provides a flow chart for data input and downstream analysis performed. First, we analyzed the overall differences between JIA subtypes, oligoarticular JIA (oligo), extend-to-be JIA (ETB), and polyarticular JIA (poly). Heatmaps reveal the 30 top variable genes which show heterogeneity within subtypes between single cells (Fig. 2B). These genes have the greatest differential expression and account for the cell dispersion between single cells within each JIA subtype. When comparing specific genes, there are 16 unique differentially expressed genes within cells from oligo, 22 unique differentially expressed genes within cells from ETB, and 15 unique differentially expressed genes within cells from poly (Fig. 2C). When comparing subtypes, there are 4 genes that overlap between oligo and ETB (Fig. 2C). Of note, FBLN1 is differentially expressed. Of the 11 genes that overlap between oligo and poly, S100A4 is differentially expressed, as well as COMP and TIMP1 (Fig. 2C). There were 6 genes that overlap between ETB and poly. From this list, COL3A1, HAPLN1, and SFRP4 are differentially expressed within single cells from each subtype (Fig. 2C).

### The most prominent cluster subpopulations of FLS co-localize together but cluster dispersion increases as the disease course becomes more severe

**Fig. 3:**
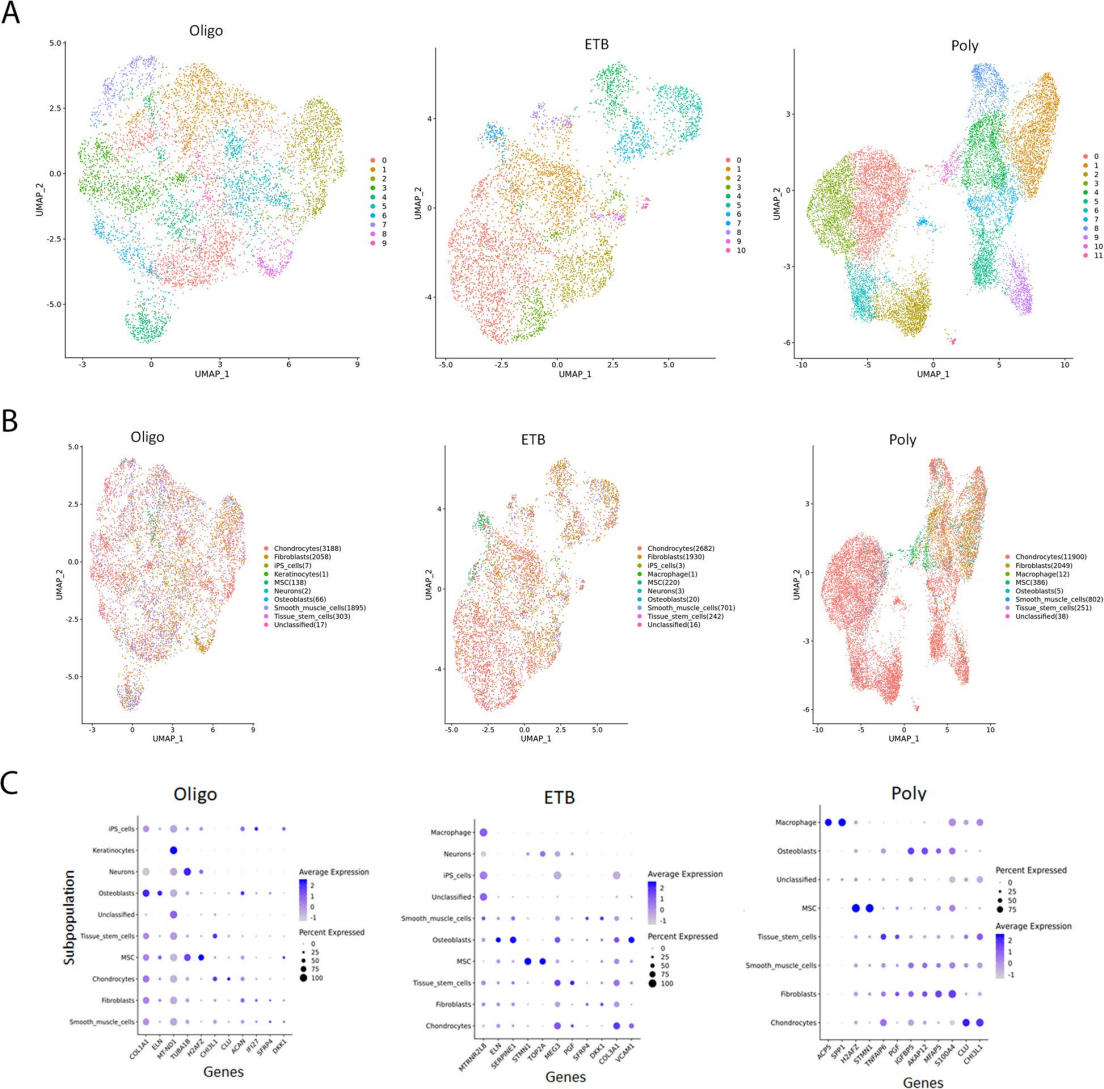
Cluster dispersion increases with disease course severity. Cells in oligoarticular mostly cluster together and begin to separate in ETB samples. The greatest dispersion is seen in the polyarticular samples (**A**). Cells were automatically annotated using the SingleR data package from Bioconductor. Shared populations between JIA subtypes included fibroblast-like cells, chondrocyte-like cells, and smooth muscle cell-like cells, as well as smaller subpopulations (**B**). Seurat single analysis identified the top genes of each projected cell type for each subtype. These genes are the highest contributors to variation among the cells. Genes that contribute the most variation among cells are as follows: oligo — COL1A1, MT-ND1, TUBA1B, ACAN, and IFI27; ETB — MTRNR2LB, SERPINE1, TOP2A, MEG3, COL3A1, and VCAM; and poly — ACP5, SPP1, TNFAIP6, IGFBP5, AKAP12, MFAP5, and S100A4 (**C**)

Each group of replicates was used as separate inputs in the Seurat data analysis package from Bioconductor. Regardless of the JIA subtype, FLS separate into distinguishable communities of cell subpopulations that vary in their gene expression. Despite a single cell type, FLS, used for initial input, the individual cells separate into various clusters of subpopulations of cells that vary in their gene expression patterns (Fig. 3A). While cells within a cluster have similar expression patterns, there are groups of cells that have different expression patterns from their neighboring clusters, demonstrating that FLS are heterogenous.

For oligoarticular JIA FLS samples, all communities co-localize together with mild separation of cluster number 4 (Fig. 3A). ETB JIA FLS samples have more separation of clusters 4, 5, and 6 while polyarticular JIA FLS samples have the most separation among their cell communities (Fig. 3A). Increases in the heterogeneity of FLS correlate to dispersion between cell clusters and this separation corresponds to progression to more severe disease courses.

### Cell clusters were identified as various subpopulations of known cell types

We used unbiased clustering since there is little known about marker genes in FLS subtypes. For validation, cells were automatically annotated using the SingleR data package from Bioconductor. Seurat single analysis was done to analyze the JIA subtypes and identify shared populations across data sets and downstream comparative analysis. Among these shared populations were fibroblast-like cells, chondrocyte-like cells, and smooth muscle cell-like cells (Fig. 3B). Other, smaller subpopulations included osteoblasts and mesenchymal stem cells (Fig. 3B).

### Identifying the top genes that account for dispersion of cells between subtypes signifies transcriptome differences between subpopulations of cells within JIA subtypes

Seurat single analysis identified the top genes of each projected cell type for each subtype. Dot plots provide visualization of the average expression of each gene in each cell type and the percentage of cells that express that gene (Fig. 3C). These genes are the highest contributors to variation among the cells. Genes that contribute the most variation among cells are as follows: oligo — COL1A1, MT-ND1, TUBA1B, ACAN, and IFI27; ETB — MTRNR2LB, SERPINE1, TOP2A, MEG3, COL3A1, and VCAM; and poly — ACP5, SPP1, TNFAIP6, IGFBP5, AKAP12, MFAP5, and S100A4 (Fig. 3C). Several of these genes encode surface and secreted proteins that have been associated with musculoskeletal and developmental diseases.

### Cells that were identified as chondrocytes are the majority cell-type subpopulation with polyarticular JIA FLS having the largest percentage of chondrocytes

**Fig. 4:**
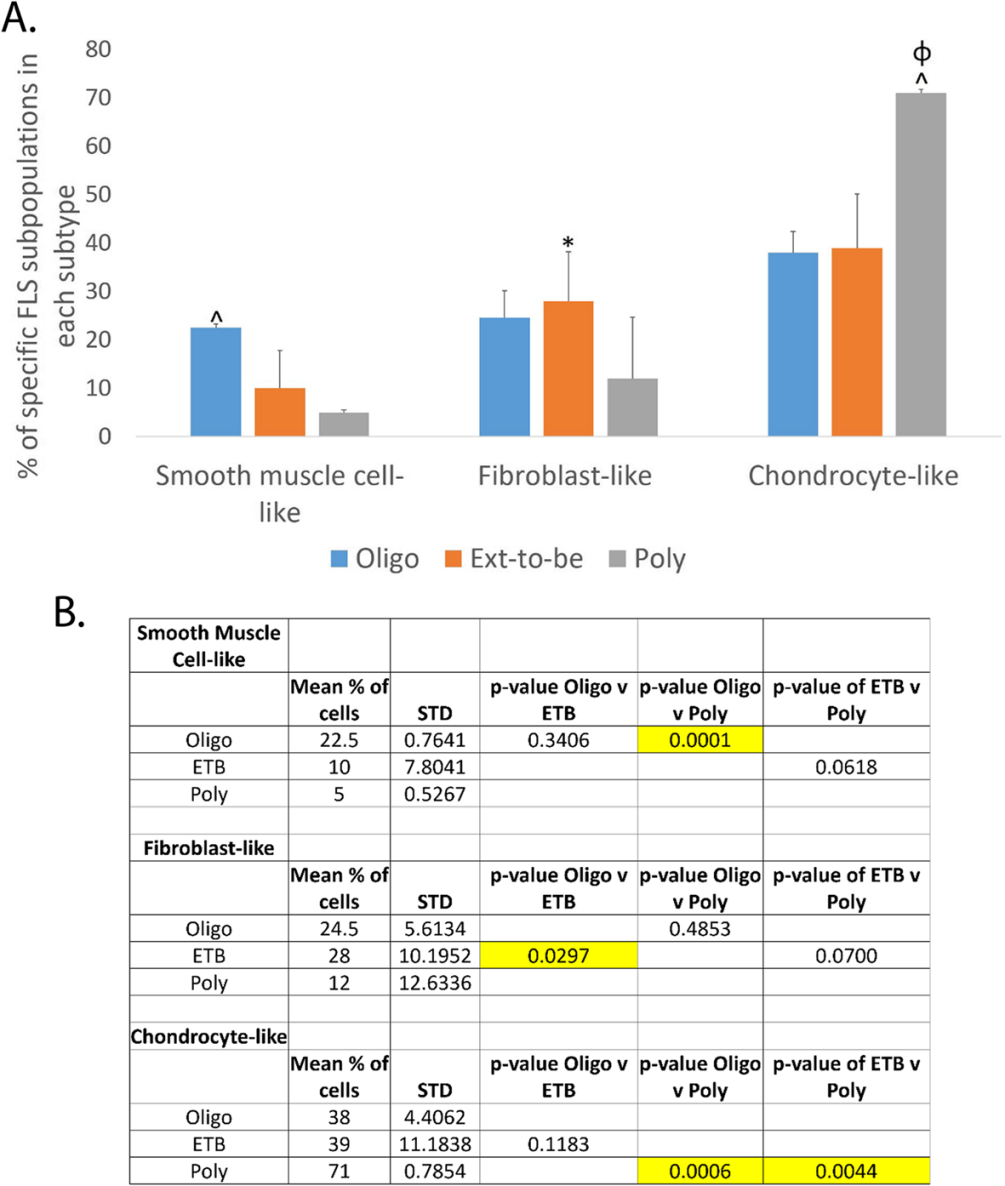
The three most populated cell-type subpopulations across JIA subtypes were identified as chondrocyte-like, fibroblast-like, and smooth muscle cell-like cells. For oligoarticular JIA FLS samples, 38% of total cells were labeled as chondrocyte-like, 24.5% of total cells represented fibroblast-like cells, and 22.5% made up the smooth muscle cell-like cell subpopulation (**A**). 39% of total cells identify as chondrocyte-like, 28% of total cells are labeled as fibroblast-like, and 10% of total cells consisted of smooth muscle cell-like cells in the groups of ETB JIA FLS samples (**A**). Lastly, polyarticular JIA FLS had 71% of total cells that were chondrocyte-like, 12% of total cells were fibroblast-like, and 5% of total cells were labeled as smooth muscle cell-like cells (**A**). There was a − 8.89 fold-decrease of smooth muscle cell-like cells between oligoarticular JIA FLS samples and polyarticular JIA FLS samples (^*p* = 0.0001) (**B**). Fibroblast-like cell percentage was significantly higher in ETB JIA FLS than oligoarticular JIA FLS (*p = 0.0297) (**B**). Chondrocyte-like cell percentage significantly increased in polyarticular JIA FLS compared to oligoarticular JIA FLS (^*p* = 0.0006) and ETB JIA FLS (φ*p* = 0.0044) (**B**)

While many cell subpopulations were identified, the three most populated cell-type subpopulations across JIA subtypes were identified as chondrocyte-like, fibroblast-like, and smooth muscle cell-like cells (Fig. 4A). As the cells evolve toward more chondrocyte-like characteristics, their fibroblast-like cell subpopulation and smooth muscle cell-like subpopulation decrease as the disease course becomes more severe, particularly between oligoarticular JIA FLS and polyarticular JIA FLS samples (Fig. 4B).

### The heterogeneity of JIA FLS persists in differential gene expression within cell subpopulations of JIA subtypes

**Fig. 5:**
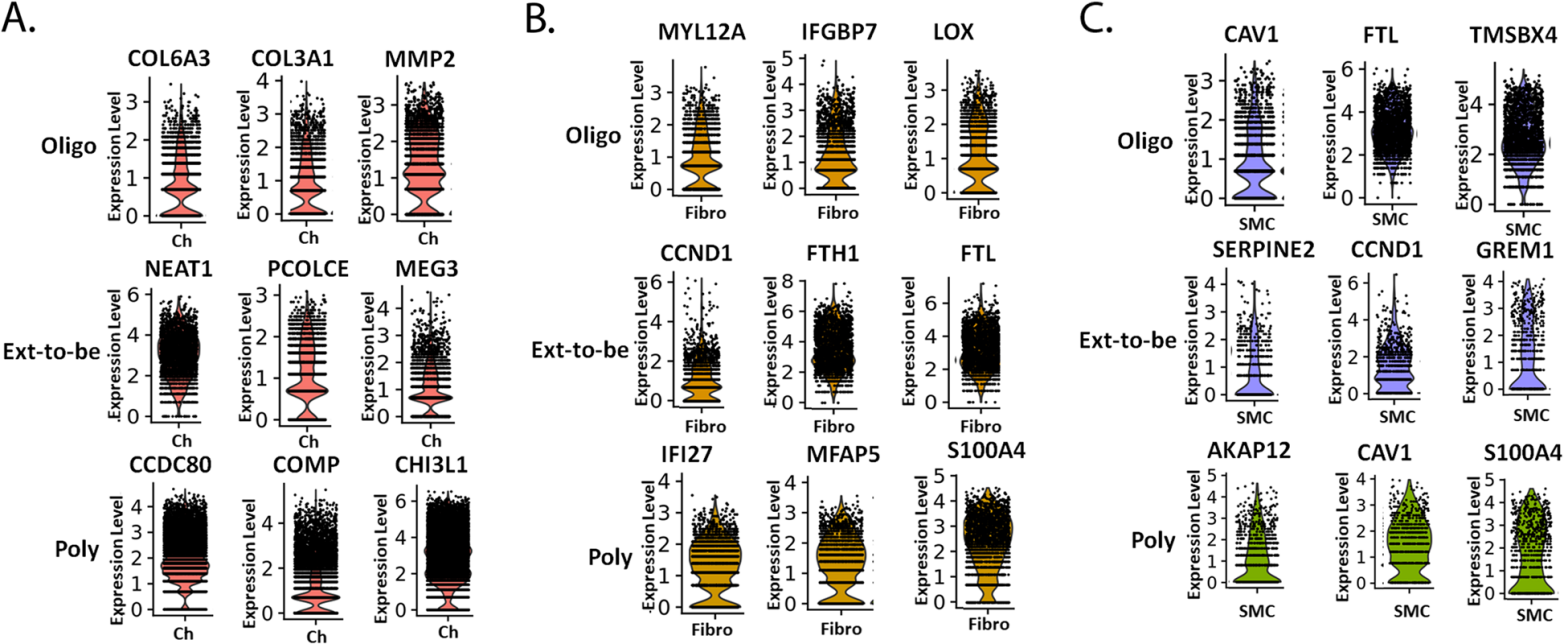
Top upregulated genes for each subpopulation. Genes expressed in chondrocytes from oligoarticular JIA were COL6A3 (65.1%), COL3A1 (66.8%), and MMP2 (85.5%) (**A**). Genes expressed in the greatest percentage in chondrocytes from ETB JIA were NEAT1 (99.2%), PCOLCE (83.7%), and MEG3 (86.7%) (**A**). Genes expressed in the greatest percentage in chondrocytes from polyarticular JIA were CCDC80 (99.7%), COMP (72.8%), and CHI3L1 (93.9%) (**A**). Genes expressed in fibroblasts from oligoarticular JIA were MYL12A (74.5%), IGFBP7 (82.9%), and LOX (77.5%) (**B**). Genes expressed in fibroblasts from ETB JIA were CCND1 (74%), FTH1 (99.8%), and FTL (99.7%) (**B**). Genes expressed in fibroblasts from polyarticular JIA were IFI27 (81.1%), MFAP5 (81.8%), and S100A4 (93.8%) (**B**). Genes expressed in smooth muscle cell-like from oligoarticular JIA FLS were CAV1 (67.5%), FTL (99.9%), and TMSB4X (98.8%) (**C**). Genes expressed in smooth muscle cell-like from extend-to-be JIA FLS were SERPINE2 (86.7%), CCND1 (79.7%), and GREM1 (58.3%) (**C**). Genes expressed in smooth muscle cell-like cells from polyarticular JIA were AKAP12 (62%), CAV1 (80.2%), and S100A4 (72.2%) (**C**). *p*-values were < 0.01

The most prominent subpopulations between JIA subtypes were the same: chondrocyte-like cells, fibroblast-like cells, and smooth muscle cell-like cells; however, transcript analysis revealed the differential expression of genes for these subpopulations between JIA subtypes. Seurat single analysis identified the top differential genes of each projected cluster or cell subpopulation for each JIA subtype (Fig. 5A). Further analysis revealed overlap among the chondrocyte-like subpopulation gene lists between JIA subtypes. By removing the overlapping genes, we identified unique genes that distinguish the chondrocyte-like subpopulations between JIA subtypes (Supplemental table 1).

When analyzing gene expression of the subpopulation of fibroblast-like cells and smooth muscle cell-like cells within the JIA subtypes, we identified unique genes that distinguish the fibroblast-like subpopulations and the smooth muscle cell-like subpopulations between JIA subtypes (Fig. 5B, C; Supplemental table 2).

Considering the chondrocyte-like cell cluster of JIA FLS is the predominant subpopulation across JIA subtypes, the expression of unique genes in these cells could distinguish between JIA subtypes. While many genes overlap, the unique genetic fingerprint of this subpopulation shows that even in a single uniform cluster of chondrocyte-like cells, there is heterogeneity between JIA subtypes. This prompted us to closely examine the transcriptome of the chondrocyte-like subpopulation within each JIA subtype.

### Further analysis of the chondrocyte-like subpopulation reveals biologically relevant evidence that contributes to the pathology of JIA

Seurat integrated analysis allows for the comparison between JIA subtypes. This analysis identifies the top differential genes of each projected cell subpopulation for each JIA subtype and allows us to determine genes that not just distinguish between subpopulations of JIA subtypes but between the JIA subtypes themselves. Positive differentially expressed genes are also calculated for each subpopulation between subtypes. This allows for the identification of differentially expressed markers across JIA subtypes.

**Fig. 6:**
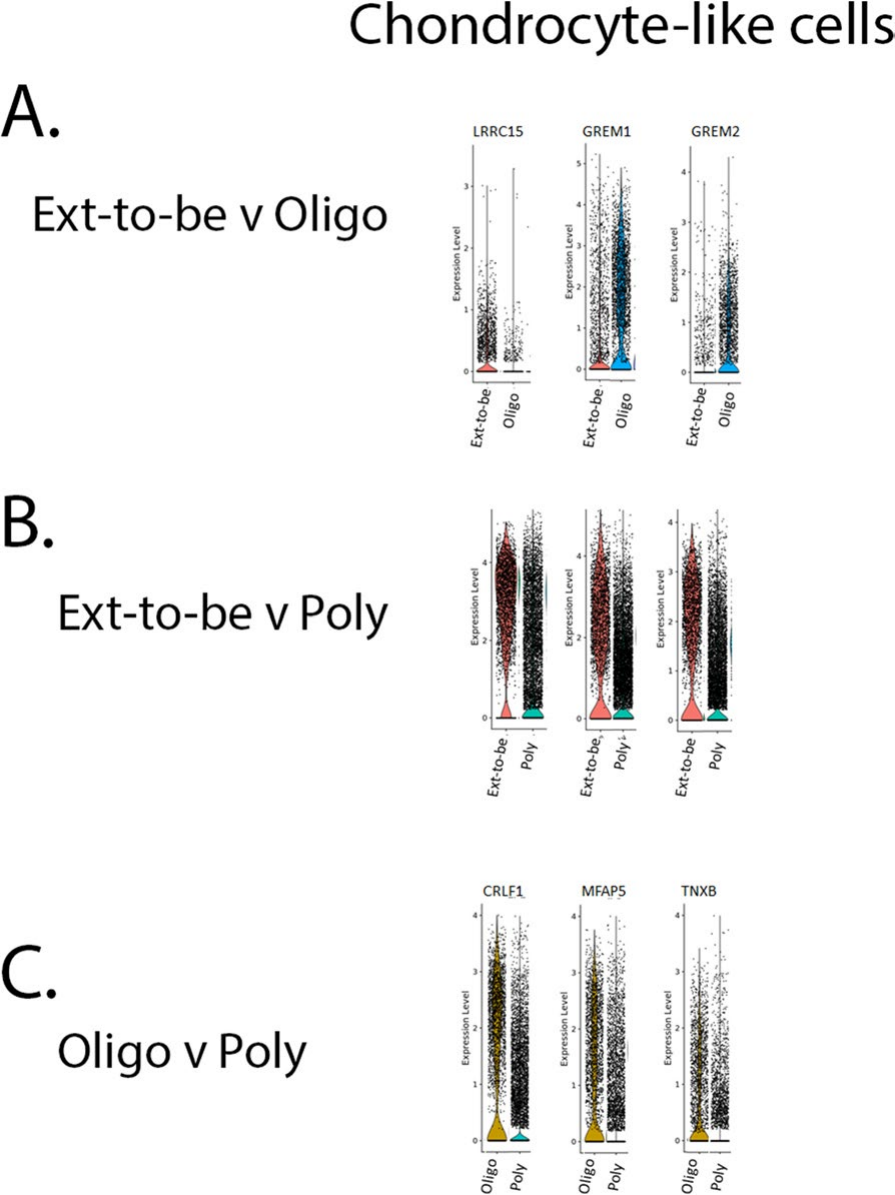
Chondrocyte-like cell subpopulation between JIA subtypes. LRRC15 is expressed in 35.8% of chondrocyte-like cells in oligo JIA FLS and 6.1% in ETB JIA FLS. 67.3% of chondrocyte-like cells in oligo JIA FLS express GREM1 compared to 34.8% in ETB JIA FLS. GREM2 is expressed in 45.8% of the chondrocyte-like cells in oligo JIA FLS compared to 11.5% in ETB JIA FLS (**A**). S100A4 is expressed in 89.6% of chondrocyte-like cells in ETB JIA FLS compared to 39% in poly JIA FLS. 75.8% of chondrocyte-like cells in ETB JIA FLS express TIMP3 compared to 48.2% in poly JIA FLS. NBL1 is expressed in 72.4% of the chondrocyte-like cells in ETB JIA FLS compared to 40.7% poly JIA FLS subpopulation from poly JIA FLS (**B**). CRLF1 is expressed in 63.1% of chondrocyte-like cells in oligo JIA FLS compared to 29.2% in poly JIA FLS. 58.7% of chondrocyte-like cells in oligo JIA FLS express MFAP5 compared to 19.7% in poly JIA FLS. TNXB is expressed in 44.6% of the chondrocyte-like cells in oligo JIA FLS compared to 11.6% in poly JIA FLS (**C**). *p*-values were < 0.01

When comparing the chondrocyte-like subpopulation from ETB JIA FLS to the chondrocyte-like subpopulation from oligo JIA FLS, there were 211 differentially expressed genes (Supplemental table 3). Of these genes, 82 are upregulated in the oligo when compared to ETB and 129 are upregulated in ETB when compared to oligo. Ingenuity pathway analysis (IPA) revealed the top diseases and disorders associated with this gene list: cancer, organismal injury and abnormality, gastrointestinal disease, and immunological disease. These diseases and disorders are associated with cell movement, cell death, cell proliferation, and cell function. The top 3 differentially expressed genes within this subpopulation were LRRC15, MTRNR2L1, and GREM2 (Fig. 6). For this comparison, LRRC15 is expressed in 35.8% of chondrocyte-like cells in oligo JIA FLS compared to just 6.1% of chondrocyte-like cells in ETB JIA FLS. 67.3% of chondrocyte-like cells in oligo JIA FLS express GREM1 compared to 34.8% of chondrocyte-like cells in ETB JIA FLS. GREM2 is expressed in 45.8% of the chondrocyte-like cells in oligo JIA FLS compared to 11.5% of chondrocyte-like cells in ETB JIA FLS.

When comparing the chondrocyte-like subpopulation from ETB JIA FLS to the chondrocyte-like subpopulation from poly JIA FLS, there were 440 differentially expressed genes (Supplemental table 4). Of these genes, 167 are upregulated in poly when compared to ETB and 273 are upregulated in ETB when compared to poly. IPA revealed the top diseases and disorders associated with this gene list: osteoarthritis, connective tissue disorders, inflammatory disease, and skeletal and muscular disorders. These diseases and disorders are associated with cell movement, cell death, cell proliferation, and cell function. The top 3 differentially expressed genes within this subpopulation were S100A4, TIMP3, and NBL1 (Fig. 6). For this comparison, S100A4 is expressed in 89.6% of chondrocyte-like cells in ETB JIA FLS compared to 39% of chondrocyte-like cells in poly JIA FLS. 75.8% of chondrocyte-like cells in ETB JIA FLS express TIMP3 compared to 48.2% of chondrocyte-like cells in poly JIA FLS. NBL1 is expressed in 72.4% of the chondrocyte-like cells in ETB JIA FLS compared to 40.7% of chondrocyte-like cells in poly JIA FLS.

When comparing the chondrocyte-like subpopulation from oligo JIA FLS to the chondrocyte-like subpopulation from poly JIA FLS, there were 266 differentially expressed genes (Supplemental table 5). Of these genes, 124 are upregulated in poly when compared to oligo and 142 are upregulated in oligo when compared to poly. IPA revealed the top diseases and disorders associated with this gene list: cancer, organismal injury and abnormality, gastrointestinal disease, and connective tissue disorders. These diseases and disorders are associated with cell movement, cell death, cell proliferation, and cell function. The top 3 differentially expressed genes within this subpopulation were CRLF1, MFAP5, and TNXB (Fig. 6). For this comparison, CRLF1 is expressed in 63.1% of chondrocyte-like cells in oligo JIA FLS compared to 29.2% of chondrocyte-like cells in poly JIA FLS. 58.7% of chondrocyte-like cells in oligo JIA FLS express MFAP5 compared to 19.7% of chondrocyte-like cells in poly JIA FLS. TNXB is expressed in 44.6% of the chondrocyte-like cells in oligo JIA FLS compared to 11.6% of chondrocyte-like cells in poly JIA FLS. Unique genetic fingerprints within the chondrocyte-like subpopulation can be used to further distinguish between JIA subtypes.

## Discussion

Despite being a predominant cell type in joint development, little is still known about the role of synovial fibroblasts in the pathogenesis of JIA. In this study, we not only reveal heterogeneity between subtypes of JIA but the differential genetic composition of subpopulations within subtypes of JIA using scRNA-sequencing. Here, we characterize FLS from oligoarticular, extended-to-be, and polyarticular JIA. We provide extensive gene signatures that describe similarities in expression to define fibroblasts and both intra-subtype and intra-subpopulation heterogeneity in gene expression that can differentiate between our subtypes of interest and the chondrocyte-like subpopulation of these JIA subtypes.

To our knowledge, this is the first study that specifically examines fibroblast-like synoviocytes isolated from patients with juvenile idiopathic arthritis using scRNA-sequencing. Although there is minimal literature on this topic in JIA, published literature does support heterogeneity of FLS in rheumatoid arthritis (RA), such as the presence of p53 mutations in a subpopulation of FLS [18] and certain FLS subpopulations have been linked to disease activity [21–23]. These prior studies emphasize the existence of heterogeneity among FLS and the importance of these cells in the invasive destructive process of RA. An examination of FLS in JIA and their association with disease progression has not been reported in the literature. Further exploration of in-depth transcriptome analyses like this one can reveal how differentially expressed genes not only contribute to the overall pathogenesis of JIA but how differences in gene expression within subpopulations of FLS can reveal comprehensive differences between JIA subtypes.

In this study, we show that JIA FLS are heterogenous and that this heterogeneity persists through several layers of gene analysis. We examined the top 30 variable genes which show heterogeneity within JIA subtypes between single cells. Of note, FBLN1, a marker of chondrocyte proliferation in osteoarthritis [28], is differentially expressed between oligoarticular FLS and ETB FLS. S100A4, a well-characterized marker of fibroblasts, is differentially expressed, as well as COMP and TIMP1 when comparing oligo and poly FLS. COMP is a marker of cartilage turnover in RA [29] while TIMP1 inhibits metalloproteases that degrade the extracellular matrix. Between ETB and poly, COL3A1, HAPLN1, and SFRP4 are differentially expressed within single cells from each subtype. Mutations in the COL3A1 gene have been linked to arthritis and bone disorders [30]. HAPLN1 has been linked to RA [31]. SFRP4 inhibits Wnt signaling, a disease regulating pathway of RA [32]. These proteins are expressed by chondrocytes and are key to cartilage development, supporting our data that chondrocyte-like cells are the predominant cell subpopulation within all JIA subtypes.

Interestingly, as disease course becomes more severe, cells have greater dispersion and cells become more chondrocyte-like. Mizoguchi et al. found that different subpopulations of FLS play distinct roles in joint destruction in RA [22]. Growth disorders are common in joints of patients with JIA. Gaspari et al. suggest that JIA FLS express pro-inflammatory proteins that can directly affect growth plate chondrocytes and lead to joint growth disturbances observed in JIA [33]. Our own work shows that JIA FLS have chondrocyte-like features [11]. This finding is mirrored in our current scRNA-sequencing study. As disease progresses toward a polyarticular course, FLS become more chondrocyte-like and less fibroblast-like or smooth muscle cell-like. FLS becoming more chondrocyte-like could contribute to joint growth disturbances observed in JIA.

Despite the conservation of cell subpopulations within JIA subtypes, we found significant transcriptome differences within the chondrocyte-like subpopulation between JIA subtypes, suggesting another layer to the heterogeneity observed in JIA FLS. Specifically, GREM1 and GREM2 were overexpressed in a greater percentage of chondrocyte-like cells from oligo JIA FLS when compared to ETB JIA FLS. GREM1 and GREM2 are BMP antagonists and members of the TGFβ superfamily, a signaling pathway that plays a critical role in chondrocyte differentiation [34, 35]. S100A4 and TIMP3 were differentially expressed between ETB and poly JIA FLS. Increased expression of S100A4 has recently been found in proliferating synovial fibroblasts and synovial tissue in patients with RA [36]. TIMP3 is a metalloprotease inhibitor, and its family members can prevent MMPs from degrading the extracellular matrix in cartilage, a characteristic of chronic rheumatic inflammatory diseases [37]. CRLF1 and MFAP5 had lower total cell percentage expression in poly JIA FLS compared to oligo JIA FLS. Repression of CRLF1 expression causes mesenchymal stem cells to differentiate as chondrocytes [38]. MFAP5 promotes cell proliferation in tumors and interacts with the ERK/MMP signaling pathways [39]. Differential expression of genes that play a role in cellular processes supports our findings found in IPA that show that the chondrocyte-like subpopulation genome promotes cell movement, cell proliferation, and cell function. While there are commonalities expected in gene expression for normal chondrocytes, like extracellular matrix-related genes, genes that regulate cartilage formation, and genes from the TGFβ superfamily, these differences could have pathogenic implications for chondrocyte-like FLS between JIA subtypes. Namely, less severe forms of JIA (oligo and prior to extension) express genes that attenuate disruption of the extracellular matrix while chondrocyte-like cells from poly JIA FLS downregulate expression of genes that are more specific to joint development and collagen formation.

The limitation of this study is that the sample number is small. Despite the sample number being limited, we found biologically relevant differences between JIA subtypes that support a critical role for FLS. We also demonstrate that the chondrocyte-like cell subpopulation can be used to distinguish between these subtypes. Specifically, ETB has a unique genetic fingerprint that can be identified prior to extension to a more severe disease course and as patients evolve to a polyarticular course, the FLS become increasingly more chondrocyte-like. scRNA-sequencing libraries such as these provide the stepping stone to future studies that further elucidate the role of FLS in disease progression and pathogenesis of juvenile idiopathic arthritis.

## Conclusion

We determined that spatially, physical cells devolve into a greater number of clusters as the disease course progresses with oligoarticular JIA FLS having the fewest cell clusters and the most overlap between clusters while polyarticular JIA FLS had the most clusters and the greatest distance between clusters. It was also discovered that annotated cell clusters revealed several cell types. These subpopulations of cells are preserved across JIA subtypes with the greatest number of cells identified as chondrocyte-like, fibroblast-like, and smooth muscle cell-like cells; however, the percentage of these subpopulations changes with disease severity. The highest percentage of total cells was identified as chondrocyte-like cells in all JIA subtypes; however, polyarticular JIA FLS had the greatest percentage of these cells, suggesting JIA FLS become more chondrocyte-like as the disease progresses. Within the subpopulation of chondrocyte-like cells, heterogeneity persists. Unique genes were expressed within each chondrocyte-like subpopulation and differed depending on which JIA subtype was being analyzed. The unique gene expression profiles of this subpopulation of cells can distinguish between the JIA subtypes.

## Supplementary Information

**Additional file 1: Supplemental Table 1.** 

**Additional file 2: Supplemental Table 2.** 

**Additional file 3:** **Supplemental Table 3.**

**Additional file 4: Supplemental Table 4.**

**Additional file 5: Supplemental Table 5.** 

## Data Availability

The datasets used and/or analyzed during the current study are available as supplemental material within this manuscript. Additional data is available from the corresponding author on reasonable request.

## Abbreviations

JIA: Juvenile idiopathic arthritis
oligo: Persistent oligoarticular JIA
ETB: Extend-to-be oligoarticular JIA
poly: Polyarticular JIA
FLS: Fibroblast-like synoviocytes
scRNA-seq: Single-cell RNA-sequencing
IPA: Ingenuity Pathway Analysis
RA: Rheumatoid arthritis
