# Supplementary material for: Single-cell analysis reveals heterogeneity of juvenile idiopathic arthritis fibroblast-like synoviocytes with implications for disease subtype
Source: Arthritis Res Ther. 2022 Sep 27;24:225. doi: 10.1186/s13075-022-02913-8 (PMC9513865; doi:10.1186/s13075-022-02913-8)
Supplement: Supplementary file 1 — Additional file 1: Supplemental Table 1. [file 13075_2022_2913_MOESM1_ESM.pdf]

Oligoarticular

*all p-values <0.00001	Gene	Log Fold Change	Chondrocyte (%)	Other cell types (%)
	DCN	0.368	89.2	85.4
	ENPP2	0.295	27.5	10.7
	PLPP1	0.292	24.1	8.9
	ZFP36L1	0.262	48.4	29.6
	EFEMP1	0.254	61.3	50.9

ETB

*all p-values <0.00001	Gene	Log Fold Change	Chondrocyte (%)	Other cell types (%)
	VCAM1	0.781	60.7	18.6
	COL3A1	0.766	85.5	67
	FBLN1	0.709	56.6	24.4
	CLU	0.645	36.7	9.3
	MEG3	0.610	86.7	63.6
	PCOLCE	0.602	83.7	56.5
	NEAT1	0.594	99.2	96.1
	STEAP4	0.592	38.4	12.9
	FRZB	0.578	19.2	4.5
	PRG4	0.558	19.8	3.5
	CYP1B1	0.557	87.5	73.6
	MALAT1	0.543	95.6	89.5
	SCRG1	0.539	43.7	13.8
	PTGDS	0.526	34.6	13.2
	PGF	0.504	33.1	16.2
	COL12A1	0.501	97	86.4
	C1R	0.497	57.5	31.5
	SAT1	0.492	53.5	33.2
	COL1A2	0.480	99.1	96.1
	ABI3BP	0.477	83.7	60.9
	MRC2	0.471	80.2	50.8
	PIEZO2	0.464	45.5	16.9
	FSTL1	0.463	97.4	90.2
	PRELP	0.448	38.6	10.5

	COL6A3	0.437	79.7	55.2
	COL6A1	0.436	95.5	79
	TIMP3	0.435	93.2	85.7
	FOS	0.433	41.3	21.8
	PENK	0.432	51.8	28.5
	POSTN	0.432	39.2	29.2
	NBL1	0.423	90.6	78.5
	COL6A2	0.421	96.5	85.6
	C1GALT1	0.420	74.6	45.2
	WNT5A	0.417	39.6	15.5
	CD9	0.407	72.7	47.1
	XIST	0.401	73.5	46.6
	ISLR	0.398	86.8	67.1
	IFITM2	0.389	68	45.8
	LRP1	0.388	88.6	70.1
	PLXDC2	0.378	36.2	11.8
	KYNU	0.367	28.1	6.4
	TNC	0.366	40.3	16.4
	SDC4	0.365	56.9	33.7
	PLEC	0.362	85.2	66.8
	SEMA3C	0.360	83.1	73.9
	LTBP3	0.357	65.3	41.1
	BZW1	0.354	82	62
	C1S	0.352	47.1	22.7
	CD164	0.347	64.9	42.8
	COL1A1	0.345	97.7	92.4
	PTGES	0.339	40.7	18
	OGN	0.338	25.2	3.5
	IFITM3	0.337	90.3	79.8
	JUNB	0.333	46.2	27.4
	GALNT1	0.332	73	51.9
	NPW	0.328	23.6	7.5
	CREB5	0.327	38	13.3
	B4GALT1	0.327	80.4	57.8

	APLP2	0.327	88	68.9
	PSAP	0.323	79.1	55.3
	TNFAIP6	0.321	30.9	14.2
	FAM118A	0.320	30.7	8.7
	BGN	0.319	69.8	50.2
	RBPJ	0.318	78.7	52.6
	ADIRF	0.316	96.2	86.1
	COL5A2	0.312	56.8	38.2
	WSB1	0.309	44.6	22.1
	AHNAK	0.308	95.1	83.4
	NNMT	0.307	87.1	74.1
	PNISR	0.307	47.3	23.2
	EEF2	0.307	98.5	95.2
	MXRA5	0.306	29.9	9.6
	DPYSL3	0.306	41.7	18.1
	SLC25A37	0.303	43.4	20.4
	GOLM1	0.303	37.1	15.6
	RSRP1	0.302	38.3	16.3
	N4BP2L2	0.300	50.9	26.6
	COMP	0.300	28.4	18
	PLD3	0.296	61.5	41.3
	SSC5D	0.294	36.1	11.6
	BSG	0.292	93.3	81.7
	SMOC2	0.290	22	4.5
	ECM1	0.288	48.2	24.6
	ARID5B	0.286	68.6	48.2
	IGFBP6	0.285	97.1	88.8
	ANKH	0.282	37.7	18.5
	SERPINH1	0.281	67.3	45.8
	DLX3	0.281	36	13.9
	KCNQ1OT1	0.280	32.7	15.8
	TXNIP	0.277	33.4	16.9
	ACKR3	0.273	29	10.2
	EBF1	0.272	32.3	11.3

	CLSTN1	0.270	38.7	16.3
	CLEC3B	0.270	34.5	14
	IER5L	0.270	38	18.7
	SLC2A12	0.268	24.8	4.6
	RUNX1	0.266	35.4	15.8
	CD248	0.265	68.6	48.9
	MAP1A	0.263	61.7	42.9
	CCDC80	0.263	85.9	77.6
	DDX17	0.263	63.2	43.5
	IGFBP5	0.261	82.3	74
	LMO4	0.260	53.8	36.2
	PYCARD	0.260	27.6	10.1
	ERRFI1	0.259	38.4	21.1
	TNXB	0.259	35.7	19
	PRUNE2	0.258	35	13.3
	HSPG2	0.258	74.9	51.3
	RPS2	0.257	99.7	98.8
	ZFP36L2	0.257	51.9	33.3
	MMP2	0.257	91	83.4
	EMILIN1	0.256	39.6	19.5
	PDE1A	0.256	29.6	8.8
	IGFBP4	0.254	98.8	96.4
	TRAM1	0.254	56.1	44.5
	FGFR1	0.253	57.9	34.5
	CHI3L1	0.253	27.2	18.7
	FKBP10	0.252	78.5	56.4
	DDX5	0.251	86.2	65.6
	RPLP0	0.250	99.2	97.5
	FGF2	-0.253	27	34
	ANKRD1	-0.254	1.3	11.3
	MTRNR2L12	-0.255	70.5	71.9
	MFAP5	-0.256	29.8	37.2
	ATP6V0E1	-0.258	72.4	70.2
	TUBA1B	-0.261	70.1	71

	SRGN	-0.262	3.7	14.5
	HINT1	-0.265	78.3	84.8
	PSG5	-0.267	1.1	14.8
	KRTAP1-5	-0.272	2.6	12.8
	MT-ND6	-0.273	57.2	65.5
	HAPLN1	-0.276	9	18.1
	CDH2	-0.281	1.4	16.9
	OAZ1	-0.282	97.1	96.4
	TPM1	-0.287	85.6	90.3
	PHACTR2	-0.291	22.8	31.5
	MAP1B	-0.303	65.4	74
	COL8A1	-0.314	15.7	26.5
	STMN1	-0.314	13	17.9
	CYR61	-0.322	25.6	34.4
	C12ORF75	-0.323	52.3	58.5
	ALCAM	-0.330	11.5	24.9
	MT-ND5	-0.331	92.5	89.1
	TXNRD1	-0.338	17	26.8
	H2AFZ	-0.339	18.2	25.1
	CITED2	-0.341	19.7	30.7
	UBE2S	-0.344	12.2	19.8
	MT-ND2	-0.352	99.9	99.4
	LDHA	-0.356	85.9	88.5
	TGM2	-0.357	5	17.8
	MT-ND4L	-0.358	72.5	74.2
	MYL12A	-0.367	83.7	88.2
	SPINK6	-0.370	4	11.6
	SCG5	-0.383	4.5	17
	KRT7	-0.388	11.1	27.2
	OSBPL8	-0.407	13.3	24.8
	CALD1	-0.413	87.4	89.9
	TNFRSF11B	-0.422	1.9	12.8
	CD59	-0.433	69.6	72
	SERPINE1	-0.441	29	40.1

	PTX3	-0.444	24.9	34.1
	HSPB7	-0.447	31.5	46.1
	F3	-0.454	6.8	18.5
	CRYAB	-0.471	77.7	85.3
	TMSB4X	-0.500	99.5	99.6
	TAGLN	-0.504	61.3	68
	SERPINE2	-0.506	77	84.4
	KRT19	-0.521	1.2	15.7
	IFI27	-0.546	38.5	56.7
	GAS6	-0.565	62.8	70.6
	THBS1	-0.589	36.9	44.1
	FTH1	-0.592	99.9	99.7
	FTL	-0.619	99.6	99.6
	CCND1	-0.627	60.6	71.4
	STMN2	-0.685	2.2	22.9
	STC2	-0.687	14.8	31.5
	MTRNR2L8	-0.733	35	43
	GREM1	-0.747	28.7	46.1
	SFRP4	-0.869	8.4	26.5
	DKK1	-1.230	9.3	36.3
Polyarticular	Gene	Log Fold Change	Chondrocyte (%)	Other cell types (%)
*all p-values <0.00001	CHI3L1	1.528	93.9	49.2
	CLU	1.080	88.1	33.3
	COMP	1.073	72.8	23.9
	MT-ND2	1.039	99.8	98.4
	MT-ND5	0.995	98	83.3
	MT-ND1	0.936	100	99.7
	MTRNR2L12	0.788	80.6	44.1
	CCDC80	0.753	97.7	79.5
	CXCL6	0.733	55.4	17.4
	CPA4	0.703	47.4	15
	EDIL3	0.689	50	10
	MT-ND4L	0.666	84	51.3

	MEG3	0.654	97.1	76.4
	FOS	0.641	90.5	51.5
	TNC	0.623	78.4	36.5
	EGR1	0.613	78.5	30.6
	RPS4Y1	0.607	65.4	16.9
	MT-ND3	0.601	100	99.7
	REV3L	0.584	59.4	24.5
	TIMP1	0.582	99.9	99.4
	RPL13A	0.576	99.8	97.9
	NDUFA4L2	0.552	96.4	73.3
	HAPLN1	0.546	39.9	15.6
	RPS20	0.532	93.7	67.8
	C1R	0.527	92.5	57.1
	PRELP	0.522	55.1	24.2
	CYR61	0.520	89.7	54.8
	FOSB	0.508	50.4	20.9
	VEGFA	0.499	56.6	22.8
	COL3A1	0.498	96.3	73.6
	COL6A3	0.497	94.9	69.8
	EPAS1	0.483	59.1	22.2
	SOD2	0.481	64	33.8
	NEAT1	0.477	99.9	98.8
	PTGS2	0.474	29.1	9
	CCNL1	0.474	70.2	34.9
	RBP4	0.464	28	7.7
	COL6A1	0.460	99.1	93.9
	MT-ND4	0.455	100	99.6
	CYTL1	0.450	29.4	11.3
	NNMT	0.446	98.6	83.7
	TGFBI	0.445	98.3	90.8
	SLC38A2	0.444	88	56.3
	MT-ND6	0.434	66.3	42.1
	WSB1	0.434	83.3	49.5
	CFH	0.433	32.6	16.3

	DUSP1	0.432	81.6	48.9
	HES1	0.427	35.3	13.5
	MT-ATP6	0.422	99.9	99.5
	PDGFRA	0.415	79.5	44.1
	ATF4	0.411	88.2	54.5
	ID2	0.401	85.1	57.4
	COL5A2	0.399	88	59.4
	C3	0.396	26.1	6.5
	PLOD2	0.395	71.8	39.7
	P4HA1	0.395	57.6	29.5
	MEST	0.394	72.5	37.5
	IER3	0.393	74	38
	RPL31	0.388	93	75.7
	COL6A2	0.386	99.7	97.6
	MT-CO3	0.376	99.6	99.4
	MT-CYB	0.374	99.9	99.6
	JUN	0.374	61.7	35
	BGN	0.368	96.7	79.5
	RPL27A	0.365	99.9	99.5
	NR4A2	0.365	28.9	8.1
	CPE	0.361	20.4	4
	COL12A1	0.361	99.2	88.5
	RPS11	0.360	99.8	99.5
	QSOX1	0.354	91	61.8
	CEBPD	0.353	71.2	39.9
	MT-ATP8	0.351	38.9	20.4
	NFKBIZ	0.344	33.3	13.1
	NR4A1	0.343	33.1	16.1
	PCOLCE	0.343	96.5	83.3
	RPL37A	0.341	100	99.9
	RPS2	0.339	100	99.9
	RPL23	0.334	99.4	97.8
	SPARC	0.329	98.5	94.5
	NAMPT	0.320	33.8	17.5

	RABGAP1	0.317	31.8	16.7
	LAMB2	0.317	85.7	57.7
	CPXM2	0.315	28.5	5.8
	IFITM3	0.310	96.2	83
	STC1	0.310	18.1	3.6
	RCAN1	0.306	42.7	25.5
	MCL1	0.303	43.3	23.8
	N4BP2L2	0.301	81.2	53
	DDR2	0.298	80.6	52.1
	VMP1	0.297	68.4	41.7
	COL14A1	0.297	26.6	8.9
	HLA-A	0.296	98.1	89
	DTWD1	0.295	51.5	32.4
	FMOD	0.291	22.3	8.3
	CHI3L2	0.286	14.2	2.6
	CA12	0.277	55.6	35.5
	EMILIN1	0.273	63.8	41.4
	DST	0.266	90.4	67.9
	PLD3	0.255	82.5	58.3
	LUM	0.253	97.9	85.3
	NDUFB6	-0.250	20	47.6
	GTF3A	-0.251	18.7	46.4
	ADRM1	-0.251	17.9	46.8
	HAS1	-0.252	2.5	17.2
	DAD1	-0.252	83.7	88.2
	MANF	-0.253	21.5	44.1
	COX7A2	-0.253	81.7	88
	POLR2E	-0.254	21.6	51.7
	POMP	-0.254	81.4	88.3
	RPA3	-0.255	11.4	32.9
	TOMM22	-0.255	15.6	44.7
	GALNT1	-0.255	49	59.9
	FGF2	-0.256	43	54
	NTAN1	-0.257	14.7	43.7

	CD81	-0.257	98.4	98.4
	SNRPG	-0.257	45.5	65.1
	HTRA1	-0.258	89.2	86.7
	BRI3	-0.258	90.5	93.1
	UQCR11	-0.258	76.6	86.6
	STOML2	-0.258	20.3	49.4
	ATP6V0E1	-0.258	87.5	90.8
	DDAH1	-0.259	11.1	35.3
	ILF2	-0.259	18	44
	HSPB6	-0.260	28.8	47.8
	EEF1A1	-0.261	100	100
	SRP9	-0.261	34.7	62.1
	TIMM10	-0.261	14.7	43.9
	EMP1	-0.261	42.1	55.4
	DBN1	-0.261	14.6	40.6
	CYCS	-0.262	25	49
	ARPC1B	-0.262	46.9	69.3
	FHL2	-0.263	78.5	80.7
	SMS	-0.263	17.9	40.3
	LSM5	-0.263	18.5	46
	CHCHD2	-0.263	88.1	93.6
	CENPW	-0.264	4.9	18.9
	ASPM	-0.264	3.7	13.2
	VAMP5	-0.264	40.8	63.6
	PSMA7	-0.264	79.2	85.1
	SERINC2	-0.264	6.6	30.9
	TPM3	-0.265	42.5	60.8
	ENG	-0.265	57.8	66
	LDHA	-0.265	96.2	95.8
	PRDX1	-0.266	92.3	95.3
	NUTF2	-0.266	14.9	44.5
	ATP5MC3	-0.267	78.2	84.9
	SUMO3	-0.267	19.1	50.2
	ATP6V0B	-0.268	28.1	55

	ITGB5	-0.268	29.3	51.5
	ACTG1	-0.268	100	99.9
	SELENOH	-0.268	19.7	52
	CCND1	-0.269	44.9	59.7
	METRNL	-0.269	66.6	74.9
	VSIR	-0.269	16.1	36.5
	SKP1	-0.270	82.1	86.8
	ANXA4	-0.270	19.6	48.9
	PSMD2	-0.270	20.3	47.5
	TMEM14B	-0.270	23.8	55.5
	FBLN2	-0.270	22.5	44.1
	VIM	-0.271	100	100
	LSM3	-0.271	31	59.1
	ITGB8	-0.271	6.5	24.5
	NEDD8	-0.271	78.2	88
	ATF5	-0.272	13.1	27.8
	TMSB10	-0.272	100	100
	TP53I11	-0.272	10.3	30.5
	NDUFS6	-0.272	51.4	73.7
	STRAP	-0.273	21.8	52.9
	DCTN3	-0.273	35.9	63.9
	SNRPD1	-0.273	20.1	45.9
	KDELR3	-0.273	22.8	50.4
	PSMA5	-0.273	21	52.1
	CARHSP1	-0.274	26.3	53.7
	CLTA	-0.274	74.9	85.4
	TMEM50A	-0.275	74.1	81
	LGALS3BP	-0.275	12.9	37.7
	UBE2M	-0.275	19.7	50.7
	FABP5	-0.275	9.9	27.1
	LAPTM4A	-0.276	85.4	88.1
	DTYMK	-0.277	6.5	26.6
	WDR1	-0.277	32.1	57
	PSMD8	-0.277	65.4	80

	PPDPF	-0.278	95.8	98.4
	NME1	-0.279	28.5	51.7
	PIN1	-0.279	15.7	46.1
	MPC2	-0.280	22	51.2
	PLP2	-0.280	57.4	70.4
	LHFPL2	-0.281	15.5	40.7
	BIRC5	-0.281	1.8	13.9
	CKS1B	-0.281	9	25.7
	PSMD7	-0.281	21	49.9
	SLC25A5	-0.281	35.5	59.7
	BRK1	-0.282	67	81.3
	TMEM160	-0.282	20.5	50.9
	GREM1	-0.282	78.8	75.5
	RECK	-0.283	24.6	49.1
	GTF3C6	-0.283	23.6	55.5
	GSTO1	-0.283	66	79.4
	MSN	-0.283	41.2	60.5
	PRIM2	-0.284	9	33.7
	LAMTOR1	-0.284	31.6	62.2
	ID1	-0.286	83.6	78.8
	PCOLCE2	-0.286	22.3	47.8
	GSN	-0.287	66.5	71.4
	MARCKS	-0.287	85.3	86.5
	GABARAPL2	-0.287	32.8	61.3
	ECM1	-0.288	18.9	46.6
	PEBP1	-0.288	75.4	86.9
	PHLDA2	-0.289	7.3	30.4
	ARPC1A	-0.289	35.2	65.3
	FBXO32	-0.289	21.3	37.8
	CSRP1	-0.289	31.2	57
	SNF8	-0.290	20.4	51.9
	CAP1	-0.295	36.8	63.1
	S100A11	-0.296	99.4	99.8
	GTF2A2	-0.296	21.7	54.8

	RAB5C	-0.296	20.6	55.6
	KPNA2	-0.297	5.4	20.5
	TSPO	-0.300	95.8	97.9
	GCNT1	-0.301	9.8	35.6
	MKI67	-0.302	0.9	11
	ITGBL1	-0.303	20.8	42.2
	PRELID1	-0.304	51.8	74.2
	PCLAF	-0.304	7	22.5
	UAP1	-0.305	23.5	49.5
	UGDH	-0.305	27.8	52.7
	COX8A	-0.305	77.8	87.3
	TXNL4A	-0.307	21.5	54.2
	HINT1	-0.308	88.7	95.2
	FABP3	-0.308	4.9	22.4
	VEGFB	-0.308	36.4	62.6
	ARPC2	-0.310	79.3	87.5
	C7ORF50	-0.311	23.9	58.1
	TYMS	-0.312	4	20.4
	DDAH2	-0.312	32.1	61.4
	EIF1AX	-0.313	56.2	75.2
	MYL6	-0.313	99.1	99.6
	CLTB	-0.315	26.3	57.7
	TNFRSF12A	-0.316	60	69.6
	PPIA	-0.317	96.7	98.5
	NDUFAF8	-0.317	32.2	64
	ITGB1BP1	-0.317	19	50.5
	NPR3	-0.318	13.7	38.7
	CALM1	-0.318	73.3	83.6
	TAGLN2	-0.318	70.8	80.7
	UQCRFS1	-0.319	19.3	53.7
	EFHD2	-0.321	17.9	47.1
	ADM	-0.321	51.4	50.2
	FLNA	-0.321	90.8	90.1
	TOP2A	-0.321	3.6	12.4

	ODC1	-0.321	22.2	50.4
	ATOX1	-0.322	64	83.4
	NDUFAB1	-0.323	26.3	61.2
	AKR7A2	-0.323	19.4	53.4
	DBI	-0.324	58.1	76.3
	DYNLL1	-0.324	90.8	96
	SH3BGRL3	-0.324	98.8	99.6
	HSPB7	-0.325	38.7	50.2
	HMGN2	-0.328	68.8	72.9
	FGF5	-0.329	7.8	24.4
	HEG1	-0.330	18.9	43.8
	CAPG	-0.330	52.8	68.3
	CEMIP	-0.330	70.9	67.3
	FRMD6	-0.331	22.8	44.4
	CD59	-0.332	83.6	89.6
	DRAP1	-0.332	82	88.9
	PDLIM7	-0.333	34.1	62.5
	MAP1B	-0.335	76.8	80.6
	PDLIM2	-0.335	77.1	85.4
	ANXA5	-0.336	97.8	98.5
	HSPB1	-0.337	78.8	88.1
	TK1	-0.338	3.1	22.1
	LMO7	-0.341	67.8	73.3
	VGLL3	-0.342	11	42.4
	DLX3	-0.343	8.5	36.8
	MYL12B	-0.343	91.4	95.9
	RHOC	-0.345	70	82.8
	LSM4	-0.345	14.9	47
	PTGES	-0.345	12.7	37.1
	NUDT1	-0.345	12.2	45.8
	CDKN3	-0.347	4.3	17.2
	SMURF2	-0.347	15.1	40.5
	YWHAQ	-0.348	55	76.4
	MYL12A	-0.349	90	94.4

	YBX1	-0.350	92.1	97
	POLR2L	-0.353	93.4	97.3
	RHOA	-0.356	77.9	88.4
	PSMB6	-0.356	38.8	69.6
	RANBP1	-0.358	21.6	49.9
	CAV2	-0.358	16.3	51.8
	HMGA1	-0.359	16.8	47.2
	H2AFV	-0.360	23.6	54.7
	BAG2	-0.360	18.8	50
	MT2A	-0.361	93.8	88
	FTH1	-0.363	100	100
	COPRS	-0.364	36.6	67.4
	LTBP2	-0.365	32.2	54.6
	YWHAH	-0.371	19.2	52.4
	SELENOW	-0.372	55.8	76.3
	RAN	-0.372	75.7	84.1
	PODXL	-0.372	1.8	19.2
	AP2S1	-0.373	84.1	92.5
	UCHL1	-0.374	16.6	39
	TUBB6	-0.378	25.6	52.2
	CRLF1	-0.379	31.4	52
	TUBA1A	-0.379	53.7	68.1
	TUBA1C	-0.381	49.6	60.5
	MTCH1	-0.382	56.3	74.7
	ANXA2	-0.383	99.1	99.5
	NBL1	-0.383	54.9	69.6
	TNXB	-0.386	9.1	28.6
	CALM2	-0.391	82.8	90.1
	ACTC1	-0.392	2.4	13.2
	LY6K	-0.395	6.5	22.4
	CCNB1	-0.396	1.9	12
	PRRX2	-0.400	47.5	68.8
	HIST1H4C	-0.401	9.1	24.1
	PTGDS	-0.401	7.5	22.9

	ACTA2	-0.402	12.4	31.7
	CKS2	-0.404	5.9	19.5
	ANPEP	-0.408	21.1	47.7
	RAB32	-0.409	26.4	60
	FN1	-0.413	99.9	99.7
	HSPA8	-0.416	76.5	87.5
	ID3	-0.417	87.7	86.7
	OAZ1	-0.425	95.3	98.4
	CALD1	-0.428	93.7	96.8
	COPZ2	-0.433	66.5	81.3
	F3	-0.433	11.9	28.8
	CENPF	-0.438	1.8	13
	ACTB	-0.440	99.5	99.5
	ACAN	-0.448	80.1	72.7
	TMSB4X	-0.451	99.3	100
	IGFBP6	-0.453	93	91.6
	PTTG1	-0.454	5.6	18.3
	S100A16	-0.455	52.5	74.5
	CLIC1	-0.456	87.6	93.9
	PPP1R14B	-0.461	82.8	91.4
	TUBB	-0.463	89.8	92.8
	THBS2	-0.463	14.5	42.8
	PENK	-0.466	22	28.1
	JPT1	-0.468	17.7	44.5
	TUBB4B	-0.469	51.8	60.1
	GUK1	-0.469	83.4	94.6
	PALLD	-0.474	24.1	62.3
	CFL1	-0.483	92.8	97
	ELN	-0.497	69.4	59
	KRT7	-0.505	13.8	29.5
	FST	-0.514	43.3	54.5
	S100A13	-0.519	82.2	91
	PFN1	-0.521	90.7	96.8
	UBE2S	-0.531	17	36.6

	MYL9	-0.531	81.7	91.5
	FGF7	-0.548	40.1	57.6
	DKK1	-0.556	6.7	23.6
	CD248	-0.556	33.2	59.9
	TAGLN	-0.568	79.5	79.4
	CXCL12	-0.581	28.7	54.4
	CRYAB	-0.586	81.7	86.1
	TPM1	-0.591	92.8	94.9
	GPX4	-0.592	85	95.4
	PGF	-0.598	13	40.9
	LOX	-0.600	87.5	88.6
	PSG5	-0.606	2.2	32.1
	TIMP3	-0.611	73.4	80.9
	C12ORF75	-0.615	46.6	75.8
	VEGFC	-0.617	33.7	66.9
	XIST	-0.629	11.8	61.5
	SCUBE3	-0.633	13.2	37.6
	H2AFZ	-0.634	37.7	53.5
	CLIC3	-0.682	20.7	55.5
	CKB	-0.684	43.1	68.9
	STMN1	-0.708	11.1	38.4
	CAV1	-0.727	74.1	88.9
	TUBA1B	-0.789	75.6	77.8
	AKAP12	-0.797	42.1	54.8
	IFI27	-0.813	26.2	65.4
	PTX3	-0.855	72.8	69.4
	MFAP5	-0.968	24.8	68.4
	S100A4	-1.010	61.3	83.3
