# Supplementary material for: Single-cell analysis reveals heterogeneity of juvenile idiopathic arthritis fibroblast-like synoviocytes with implications for disease subtype
Source: Arthritis Res Ther. 2022 Sep 27;24:225. doi: 10.1186/s13075-022-02913-8 (PMC9513865; doi:10.1186/s13075-022-02913-8)
Supplement: Supplementary file 2 — Additional file 2: Supplemental Table 2. [file 13075_2022_2913_MOESM2_ESM.pdf]

Fibroblast
Oligoarticular
*all p-values <0.0001

Gene	Log Fold Change	Fibroblast (%)	Other cell types (%)
ACAN	0.313	51.4	45.1
IFI27	0.297	32.1	18.7
LOX	0.290	77.5	63.4
PSG5	0.281	18.5	4.9
IGFBP7	0.270	82.9	77.2
MYL12A	0.269	74.5	65.2
CEMIP	0.264	49.9	41.7
CRYAB	0.253	72	57.9
PCOLCE	-0.253	58.3	65.7
SOD2	-0.258	12	23.3
TNC	-0.264	11.4	24.3
NEAT1	-0.270	92.4	92.9
PENK	-0.272	15.5	23.8
WNT5A	-0.304	13.7	25.3
COL3A1	-0.311	51.1	61
MEG3	-0.329	53.8	61.5
MMP2	-0.332	77.1	82.3
MT2A	-0.348	65.7	76.6
COL6A3	-0.376	41.5	59.3
TNFAIP6	-0.428	7.5	20.2
CLU	-0.621	8.7	25.4
CHI3L1	-1.340	34.2	51.1

ETB
*all p-values <0.0003

Gene	Log Fold Change	Fibroblast (%)	Other cell types (%)
DKK1	0.719	37.3	17.2
SFRP4	0.630	27.4	13.5
FTL	0.537	99.7	99.6
FTH1	0.531	99.8	99.8
STC2	0.522	31.8	19.8
GREM1	0.504	48.1	33.2
STMN2	0.492	25.9	7.1
IFI27	0.482	61.8	41.6
CCND1	0.429	74	62.7
TMSB4X	0.392	99.7	99.4
GAS6	0.385	73.1	64
THBS1	0.378	45.9	38.3
PTX3	0.361	35.3	27.1
F3	0.352	19.8	9.7
MT-ND5	0.339	89.7	91.1
TNFRSF11B	0.339	13.1	5.1
MTRNR2L8	0.336	43.9	37

Smooth Muscle Cell
Oligoarticular
*all p-values <0.0001

Gene	Log Fold Change	SMC (%)	Other cell types (%)
DKK1	0.506	20.4	10.4
SFRP4	0.383	21.7	16
TMSB4X	0.314	98.8	98.8
FTL	0.270	99.9	99.8
CAV1	0.259	67.5	64
CCND1	0.254	49.6	44
COL6A3	-0.251	51.1	55.7
ACAN	-0.256	42.6	48.2
ELN	-0.263	18.5	31
COL3A1	-0.264	55.4	59.3
MEG3	-0.274	56.6	60.3
COL1A1	-0.274	88.6	90.2
COL6A2	-0.278	82.3	85.9
C1R	-0.284	50.7	57.6
NEAT1	-0.285	90.3	93.5
WNT5A	-0.289	12.8	25.3
SCRG1	-0.291	6.8	20.7
ENPP2	-0.295	4.9	21.9
PCOLCE	-0.296	57.9	65.7
VCAM1	-0.297	5.2	20
BGN	-0.315	18.9	36.3
TNC	-0.317	9	24.8
FOS	-0.341	19.9	42.2
PENK	-0.351	13	24.4
CLU	-0.360	14.2	23.1
TNFAIP6	-0.389	7.7	19.8
POSTN	-0.423	58.6	60.7
MALAT1	-0.448	91.3	94.3
COMP	-0.574	8.7	26.1

ETB
*all p-values <0.0001

Gene	Log Fold Change	SMC (%)	Other cell types (%)
DKK1	0.728	42.7	21.3
GREM1	0.640	58.3	35.3
CCND1	0.633	79.7	64.6
SERPINE2	0.598	86.7	80.2
SFRP4	0.597	36.8	15.6
KRT19	0.554	22.7	7.2
SERPINE1	0.529	47.8	33.2
FTL	0.511	100	99.6
FTH1	0.494	99.6	99.8
STC2	0.482	38.7	21.8

MT-ND2	0.333	99.6	99.6
MT-ND4L	0.317	77.2	71.6
HSPB7	0.312	48.5	34.9
TXNRD1	0.310	27.9	19.5
TAGLN	0.303	70.2	62.3
CRYAB	0.297	88.9	78.3
CITED2	0.296	31.9	22.6
MT-ND6	0.292	68.4	58.4
CALD1	0.290	92.5	86.9
OSBPL8	0.288	24.6	17
KRT19	0.280	15.8	5.7
KRTAP1-5	0.278	14.8	4.8
TGM2	0.278	17.6	9.1
CD59	0.274	72.4	70.1
SCG5	0.272	17.4	8.2
MFAP5	0.266	37.7	31.8
KRT34	0.262	10.3	3.3
C12ORF75	0.259	59.2	53.9
SERPINE2	0.256	86.5	78.2
KRT7	0.251	27.2	16.1
JUNB	-0.253	26.3	40.8
ERRFI1	-0.253	17.6	34.7
OGN	-0.255	2.6	18.9
ANKH	-0.255	15.5	33.3
AHNAK	-0.255	84.5	90.9
LRP1	-0.255	73.8	81
COL1A1	-0.260	93.7	95.4
MAT2A	-0.261	19.9	36.5
COL5A2	-0.261	37.8	51.2
PNISR	-0.261	21	40.9
PITX1	-0.262	35	50.6
ECM1	-0.264	21.6	42.3
RSRP1	-0.266	14.2	32.5
DPYSL3	-0.270	14.4	36.3
NPW	-0.271	6	19.3
LTBP3	-0.273	42.6	57
IFITM2	-0.276	46.6	60.7
PTGES	-0.277	16.1	34.6
FUS	-0.278	21.8	43.8
SLC25A37	-0.279	17.9	37.5
PLXDC2	-0.280	11.3	28.9
WSB1	-0.280	19.6	38.9
ISLR	-0.281	69.9	79.2
FOS	-0.282	23.5	34.4
APLP2	-0.283	69	82

GAS6	0.480	74.5	66
HSPB7	0.462	55.5	37.2
CRYAB	0.459	87	81.1
CD59	0.454	77.6	69.9
THBS1	0.450	48.8	39.7
STMN2	0.447	26.1	11.6
TMSB4X	0.403	99.6	99.5
UCHL1	0.389	36.5	25.3
MAP1B	0.387	75.9	69.2
SPINK6	0.384	15.1	7.2
CALD1	0.376	90.3	88.5
PTX3	0.375	38.7	28.6
MYL12A	0.365	87.9	85.9
OSBPL8	0.365	32.1	17.8
PHACTR2	0.363	42.5	25.4
TAGLN	0.360	68	64.5
SCG5	0.358	23.8	9.5
SLC14A1	0.352	23	9.1
IFI27	0.345	59.9	46.7
SOD2	0.345	27.1	18.6
F3	0.342	21.3	12
NDUFA4L2	0.325	74.3	57.9
FKBP1A	0.323	64.1	53.1
HAPLN1	0.320	23.4	12.6
ALCAM	0.319	31.1	17
KRT7	0.318	34.4	17.8
ATP6V0E1	0.314	74.6	70.7
NEK7	0.313	37.4	28.8
OAZ1	0.309	97.4	96.6
SRGN	0.309	22.4	7.8
MXRA7	0.308	58.8	51.7
SKP1	0.301	67.8	60.6
C12ORF75	0.298	60.9	54.9
TGM2	0.297	23.8	10.3
LDHA	0.294	89.3	87
TNFRSF11B	0.287	17.4	6.4
CDH2	0.286	24.1	7.8
COL8A1	0.280	30.8	20.2
FGF2	0.278	40.1	29.5
KRT34	0.277	15.1	4.3
SELENOM	0.271	83	83.1
KRTAP2-3	0.269	16	5.8
HINT1	0.266	83	81.6
MYDGF	0.266	70.2	66.6
CITED2	0.256	35.1	24.4

GALNT1	-0.292	51.7	66.6
NBL1	-0.293	80.5	85.9
MT1E	-0.294	43.1	55.5
SDC4	-0.296	32.1	50.5
CD164	-0.298	41.6	58.6
BZW1	-0.300	60.6	76.5
CRIP2	-0.301	23.3	47.5
CHI3L1	-0.302	18.2	24.8
KYNU	-0.309	5.1	22
CREB5	-0.312	10.3	31.8
PLEC	-0.316	68	79
TNC	-0.317	13.7	34.2
MT2A	-0.321	91.2	93.1
TNFAIP6	-0.328	11.6	27
C1GALT1	-0.332	46.2	65
LMO4	-0.335	32.7	50.1
MRC2	-0.338	52.4	70.3
POSTN	-0.339	27.7	36.9
COL6A2	-0.344	86.8	92.5
TIMP3	-0.349	86.6	90.4
XIST	-0.352	48.3	64.2
C1R	-0.354	32.7	48.8
PTGDS	-0.367	13.1	28.1
CYP1B1	-0.369	78.2	80.9
PRELP	-0.371	8.9	30.6
COL12A1	-0.372	88.1	92.9
COL1A2	-0.374	96.5	98
CD9	-0.380	44.9	65.8
COL6A1	-0.389	78.9	90.4
SAT1	-0.391	33.7	46.9
SCRG1	-0.403	13.1	34.7
FSTL1	-0.404	90.9	94.8
WNT5A	-0.414	11.9	33.9
COL6A3	-0.416	55.1	72.1
PIEZO2	-0.418	13.2	38.4
ABI3BP	-0.421	61.3	76.4
PRG4	-0.461	2.5	15.2
PENK	-0.462	27.6	45
PGF	-0.477	13.1	29.4
PCOLCE	-0.479	57.5	74.8
FRZB	-0.485	3	15.4
NEAT1	-0.513	97.1	97.7
STEAP4	-0.525	9.9	32
CLU	-0.540	7.8	29
MALAT1	-0.556	88.9	94

CLIC4	0.251	74.5	68.7
RAB13	0.251	61.6	51.5
TNC	-0.252	15.1	29.1
SAT1	-0.252	35.8	43.5
PENK	-0.255	27.1	40.9
COL5A2	-0.260	34.1	48.5
ABI3BP	-0.260	58.9	73.1
COL1A1	-0.267	87.6	95.8
MXRA5	-0.271	4.4	20.9
NBL1	-0.275	77	85
LMO4	-0.275	33.4	45.8
IFITM2	-0.277	43.9	57.7
ISLR	-0.278	61.1	78.2
C1GALT1	-0.281	40.8	61.2
CYP1B1	-0.301	73.5	80.9
LRP1	-0.305	65.2	80.5
XIST	-0.308	39.5	61.6
FSTL1	-0.311	88.6	94.2
COL6A3	-0.314	50.9	68.6
WNT5A	-0.323	11.4	28.7
PRELP	-0.328	7	25.7
COL12A1	-0.330	81.6	92.7
MALAT1	-0.335	87	93
COL1A2	-0.342	94	97.9
COL6A1	-0.349	73	88.4
C1R	-0.358	29.7	45.4
COL6A2	-0.366	79.2	92.2
NEAT1	-0.374	93.4	98.1
PIEZO2	-0.374	12.1	32.5
PGF	-0.380	12.8	25.5
STEAP4	-0.387	10.8	26.6
PRG4	-0.391	0.9	12.4
MRC2	-0.393	45.1	67
SCRG1	-0.399	11	29.8
FRZB	-0.405	2.4	12.5
CLU	-0.416	8.3	23.8
COMP	-0.419	11.8	24.3
MEG3	-0.456	58.6	76.4
FOS	-0.471	12.1	33.4
POSTN	-0.472	27.8	34.7
PCOLCE	-0.483	48.9	71.8
FBLN1	-0.485	18.8	42.1
VCAM1	-0.487	19.1	40.6
PTGDS	-0.498	7.6	25.2
COL3A1	-0.614	59.5	77.8

MEG3	-0.563	66	78.3
FBLN1	-0.606	23.2	47.2
COL3A1	-0.619	69.1	78.8
VCAM1	-0.726	14.6	49.6

Polyarticular
*all p-values <5.56*10^8

Gene	Log Fold Change	Fibroblast (%)	Other cell types (%)
S100A4	1.072	93.8	62.1
MFAP5	1.071	81.8	27.6
PTX3	1.013	77.5	71.1
AKAP12	0.959	61.9	42.4
IFI27	0.948	81.1	28.2
SFRP4	0.859	40.9	8.2
CLIC3	0.837	69.8	22.4
IGFBP5	0.777	65.7	70.4
SERPINE1	0.771	51.3	21.9
FGF7	0.753	71.8	39.8
PSG5	0.737	42.8	3.9
LOX	0.728	95	86.6
TIMP3	0.713	90	72.8
XIST	0.698	75.3	15.2
VEGFC	0.696	76	36
GPX4	0.676	98.9	85.6
CD248	0.671	67.8	35
CKB	0.668	73.9	45.2
C12ORF75	0.668	82.8	48.8
PGF	0.667	47.9	15.1
CRYAB	0.659	93.4	81.1
CAV1	0.657	94	75
ELN	0.656	64.4	67.4
S100A13	0.652	97	82.3
PI16	0.640	11.5	2.6
TPM1	0.638	95.4	93
MYL9	0.625	96.3	82.1
SEMA3C	0.609	75.7	71.9
THBS2	0.605	54.3	15.9
SCUBE3	0.602	43	15.1
TAGLN	0.582	80.4	79.4
IGFBP6	0.572	95.7	92.2
ID3	0.568	92.8	86.7
CXCL12	0.562	58.6	30.9
ACAN	0.558	79.9	78.1
COPZ2	0.542	89.8	66.9
PTGDS	0.537	30.4	8.1
CRLF1	0.526	62.4	32.1

Polyarticular
*all p-values <0.031

Gene	Log Fold Change	SMC (%)	Other cell types (%)
IGFBP5	0.605	66.7	70
AKAP12	0.554	62	44.1
SCUBE3	0.529	30.9	18.1
S100A4	0.516	72.2	66
PENK	0.503	30.3	23
MFAP5	0.500	52.2	33.9
DKK1	0.480	21.9	10
CAV1	0.399	80.2	77.4
IFI27	0.395	52.4	34.2
SEMA3C	0.394	72.9	72.4
FST	0.381	55.9	45.3
IGFBP6	0.380	91.1	92.8
SERPINE1	0.354	38.7	25.1
TMSB4X	0.350	100	99.4
CXCL12	0.343	45.1	34
TNXB	0.340	29.8	12.7
LOX	0.323	84.7	87.9
ADIRF	0.323	90.4	85.3
MYL9	0.315	89.2	83.7
TAGLN	0.310	79.3	79.5
CKB	0.308	61.2	48.3
UCHL1	0.307	39.7	20.7
S100A16	0.301	69.1	56.9
GPX4	0.293	91.5	87.2
TIMP3	0.290	70.6	75.3
SH3BGRL3	0.289	99.5	99
S100A13	0.287	85	84.2
IGFBP2	0.283	24.4	16.1
C12ORF75	0.281	63.2	52.8
HSPB6	0.279	51.5	32.2
GUK1	0.279	89.7	85.8
CCND1	0.267	54	47.9
COPZ2	0.264	73.1	69.7
HMGA1	0.264	42	22.7
POLR2L	0.264	97	94.2
CITED2	0.256	52.1	45.3
IGFBP7	0.254	96.8	97.7
SRGN	0.253	28.6	16.5
S100A6	0.252	100	100
XIST	0.251	40.4	22.3
CXCL1	-0.250	18.8	25.6
ISLR	-0.251	60.5	83.5

NBL1	0.525	80.5	54.9
PALLD	0.515	69.7	27.2
MTCH1	0.512	83.8	57
F3	0.509	33.5	13.1
CEMIP	0.502	77.2	69
FN1	0.492	100	99.9
ADM	0.483	55.6	50.4
S100A16	0.483	78.5	54.3
CALD1	0.480	97.7	93.9
GUK1	0.480	97.8	84.2
GREM1	0.475	84.4	77
OAZ1	0.474	99.7	95.4
ACTC1	0.474	16.2	3.1
ACTA2	0.471	34.7	14.1
LTBP2	0.462	62.1	33.6
KRT7	0.458	30.6	15.4
PRRX2	0.457	75.8	48.8
TNXB	0.448	34.1	10.4
HSPB7	0.446	58.8	38.7
FTH1	0.445	100	100
RAB32	0.441	66.2	29.2
PFN1	0.440	97.9	91.2
SELENOW	0.439	83.5	57
BAG2	0.432	57.7	21.1
DKK1	0.428	23.5	8.6
PDLIM2	0.426	91.3	77.1
ANXA5	0.423	99.2	97.7
DLX3	0.420	46.3	10.2
HEG1	0.418	52.8	20.3
PTGES	0.414	46.2	14
NPR3	0.409	47.8	15.1
LMO7	0.408	79.5	67.5
UCHL1	0.404	42.7	18.5
GSN	0.402	81.8	65.4
VGLL3	0.402	51.1	13.2
HSPB1	0.397	92.7	79.1
FGF2	0.397	63.4	42.8
CLIC1	0.396	96.9	87.8
TMSB4X	0.390	100	99.4
HTRA1	0.388	93.5	87.9
FST	0.386	55.2	44.4
POLR2L	0.384	98.3	93.7
MMP2	0.384	90.6	87.8
FRMD6	0.381	48.5	24.6
MYL12A	0.380	97	90.1

FMOD	-0.258	7.2	19.7
RCAN1	-0.258	26.3	39.5
CYTL1	-0.259	17.1	25.7
JUN	-0.262	38.8	56.5
HIF1A	-0.267	52.4	76.7
PLOD2	-0.268	43.8	65.6
C3	-0.278	9.6	22.2
COL6A3	-0.280	77.3	89.8
VEGFA	-0.280	31.9	49.7
CYR61	-0.282	61.7	82.8
ID2	-0.284	60.8	79.7
CFH	-0.291	21.3	29.3
NNMT	-0.295	88.3	95.5
DUSP1	-0.295	52	75.3
HAPLN1	-0.301	24.4	34.8
EGR1	-0.304	42.6	68.9
MEG3	-0.308	86.5	92.6
REV3L	-0.314	35	52.3
CCDC80	-0.323	87.5	93.8
PTGS2	-0.326	13.5	25.1
IER3	-0.326	41.6	67.1
MT-ND4L	-0.330	61.2	77.3
TIMP1	-0.335	99.3	99.8
COL5A2	-0.339	61.8	82.5
SAT1	-0.343	60.2	77.9
C1R	-0.350	65.6	85.4
ENPP2	-0.354	20.4	42.2
BGN	-0.364	80	93.4
FOS	-0.372	55.6	82.9
TNFAIP6	-0.390	52.6	77
MT-ND1	-0.397	99.6	100
RBP4	-0.400	7.5	24.2
SOD2	-0.407	37.7	58.1
MTRNR2L12	-0.417	55.6	73.1
PRELP	-0.422	27.1	49.2
CXCL6	-0.429	26.1	47.8
EDIL3	-0.433	21.7	41.9
NDUFA4L2	-0.460	81.9	91.6
MT-ND5	-0.472	90.1	94.9
MMP3	-0.491	2.7	12.1
MT-ND2	-0.510	98.9	99.5
TGFBI	-0.519	92.4	96.8
TNC	-0.572	33.4	70.7
SCRG1	-0.588	34.9	69.1
COL3A1	-0.627	79.1	91.7

FABP3	0.376	27.4	6
ANPEP	0.376	49.9	23.7
PPP1R14B	0.376	93.8	83.4
SMURF2	0.375	45	17.3
AP2S1	0.375	95.5	84.6
FBXO32	0.373	44.9	22.1
PDLIM7	0.367	67.4	36.5
ITGB8	0.364	32	7.4
DDAH2	0.364	68.9	34.2
CFL1	0.364	97.7	93.1
HSPA8	0.360	91	77.2
MYL12B	0.360	98	91.6
VEGFB	0.359	70.1	38.2
ITGBL1	0.359	49.6	22.1
ID1	0.358	84.3	82.3
LAPTM4A	0.357	93.4	84.9
FBLN2	0.354	52.7	23.6
ATOX1	0.354	88.2	65.4
CAV2	0.353	56.5	19.5
PCOLCE2	0.352	54.3	24.2
PSMB6	0.350	73.6	41.7
VSIR	0.347	45.4	17
LBH	0.347	55.9	29.6
CD81	0.345	99.7	98.2
AKR7A2	0.344	58.3	22.5
PRIM2	0.344	40.6	10.7
RHOA	0.344	91.5	78.6
ACTB	0.343	99.5	99.5
SMOC2	0.343	27	6.9
ITGB5	0.343	59.6	30.5
ISG15	0.340	30.8	12.6
METRNL	0.339	82.6	66.4
SPON2	0.337	28.8	6.3
44815	0.336	81.2	71.8
IGFBP7	0.335	98.9	97.5
LGALS3BP	0.334	44.9	14.6
B2M	0.333	99.9	99.7
RECK	0.332	55.4	26.3
TUBA1A	0.332	69.5	55.1
SELENOP	0.332	32.3	11.4
VAMP5	0.331	71	42.2
TP53I11	0.330	36.5	11.6
EEF1A1	0.330	100	100
CLTB	0.329	62	29.1
FNDC1	0.329	34.4	13.1

CLU	-0.660	50.2	76.9
COMP	-1.052	22.6	63.7
CHI3L1	-1.198	66.3	84.6

COPRS	0.329	71.1	39.5
FHL2	0.328	86.1	77.9
CAPG	0.328	72	54
ATF5	0.328	30.8	14.2
PENK	0.326	30.7	22.3
ECM1	0.326	53.7	20.9
CD59	0.325	93.5	83.7
ANXA4	0.324	57	21.6
GABARAPL2	0.323	67.5	35.1
TUBA1B	0.322	77.3	75.9
RPS4X	0.322	100	99.9
MXRA8	0.321	93.9	85.6
GCNT1	0.320	40.1	12
RRAS	0.320	82.6	66.1
ANXA2	0.320	99.7	99.1
EIF1AX	0.318	79.4	57.6
NDUFAB1	0.317	65.4	29.6
ENG	0.316	70.9	58
ATP6V0E1	0.316	95.7	87.1
CYB5R3	0.315	95.3	85
PEBP1	0.312	91.3	76
TSPO	0.312	98.7	95.9
PXDC1	0.311	44.6	17.2
PLPP3	0.309	79	84.6
NUDT1	0.307	48.6	15.6
EPDR1	0.307	35.5	6.5
MYL6	0.306	99.7	99.1
MYO1E	0.306	44.6	14.8
SH3BGRL3	0.306	99.8	98.9
LHFPL2	0.306	45.1	17.7
FLNA	0.305	91.3	90.5
UQCR11	0.305	91.1	77.1
PDE5A	0.304	34.4	11
ISCU	0.304	68.9	44.2
PPDPF	0.303	99.2	96
CALM2	0.303	93	83.2
PODXL	0.303	19.7	3.7
TMEM50A	0.302	86	74.1
YWHAQ	0.302	80.7	56.7
S100A11	0.300	99.9	99.4
MAP1B	0.299	82.2	77
HSPB6	0.299	51.4	30.4
POSTN	0.299	51.3	71.8
ITGAV	0.298	54.7	26.4
BRK1	0.298	85.9	67.9

RAB5C	0.297	59.7	23.9
KANK2	0.297	51.5	21.5
ETHE1	0.295	44.6	14.3
NDUFAF8	0.294	66.5	35.3
HSPA1A	0.293	44.9	18.8
YBX1	0.293	98	92.5
VAT1	0.292	72.1	43.9
GPNMB	0.292	46.2	22.8
EPHX1	0.291	50.8	33.2
BRI3	0.291	95.7	90.4
OLFML3	0.290	46.8	21.4
RHOC	0.290	85.7	71
NPW	0.290	21	10.9
HMGA1	0.289	47.3	20.1
PPIA	0.288	99.2	96.8
GALNT1	0.287	62.5	49.8
CHPF	0.287	66.1	46.7
HINT1	0.287	97.5	89.1
DSTN	0.286	99.4	98.9
RPS27L	0.286	99	95.4
UQCRFS1	0.285	57.3	22.5
LAMTOR1	0.284	66.3	34.4
EIF3F	0.283	93.6	85.5
DBN1	0.283	45.3	16.8
DYNLL1	0.283	97.8	91.1
OST4	0.283	98.6	95
C16ORF45	0.283	43.3	15.6
SKP1	0.283	91.1	81.9
ACKR3	0.282	33.4	13.1
COX7A1	0.281	53.8	26.4
MPC2	0.281	54.5	24.8
TIMP2	0.280	95.9	91.6
C7ORF50	0.280	59.4	27.5
RPS10	0.277	96	88.7
RAB23	0.276	38.1	12.3
OAF	0.275	38.3	11.8
EMP1	0.275	57.3	43.3
YWHAH	0.274	52.1	23
DAD1	0.274	91.5	83.7
MAP1A	0.274	64.2	39.4
GSTO1	0.273	82.9	66.9
GTF2A2	0.272	58.3	24.9
HAS1	0.272	18.6	3.9
KAZALD1	0.272	40.8	14.4
PRDX1	0.271	97.5	92.3

OSR1	0.270	24.5	5.8
PTGIS	0.270	28.8	8.8
TXNL4A	0.270	55.4	25
CLTA	0.269	89.7	75.4
ADD3	0.269	46.4	20.8
PHF20	0.269	47.8	19.5
CARHSP1	0.269	56.5	28.9
SCG5	0.269	26.2	10
PRUNE2	0.269	43.1	20.7
SIPA1L1	0.268	54	24.6
NEDD8	0.268	91	78.8
KDELR3	0.268	52.5	25.6
YPEL3	0.267	57.4	33.7
ADAMTSL1	0.267	43.4	16.7
ZFP36L2	0.266	67.1	54
DCTN3	0.266	67.6	38.4
UAP1	0.265	49.4	26.5
SNF8	0.265	54.3	23.6
PDGFRB	0.264	41.5	19.2
FBLN5	0.263	34.4	12.8
SERINC2	0.262	34.8	8.7
TXN	0.262	99.5	97.3
VCAN	0.262	69.6	62.1
FLRT2	0.261	33.5	13.6
FAM89B	0.261	48.9	20.4
CXXC5	0.261	61.4	37.9
PDE1A	0.261	32	8.2
PSMD8	0.260	83.6	66.5
LARP6	0.260	57.6	29
ABI3BP	0.260	53.5	44.8
NTAN1	0.260	47.5	17.4
FLNC	0.260	32.3	11.6
SELENOH	0.259	55.4	22.8
SRI	0.259	71.9	52.1
UGDH	0.259	52.5	30.7
MARCKS	0.259	88.6	85.1
PODN	0.259	29.1	8.3
SNX3	0.258	85.3	70.5
ACTG1	0.258	100	100
SUMO3	0.258	53.5	22
CSRP1	0.257	58.6	33.8
RSU1	0.257	48.1	18.1
CAP1	0.257	65.2	39.5
ATP5F1E	0.257	98.7	94.5
RNF187	0.257	46.8	17.8

DDAH1	0.256	37.4	13.4
NDUFB5	0.255	53.9	23.4
ARL2	0.255	69.8	45.3
BEX3	0.255	84.5	72.3
ARPC1A	0.254	66.5	38.4
CALU	0.254	95.9	93.3
ATP6V0B	0.254	58.5	30.6
ATP5MC3	0.253	88.5	78.4
SBDS	0.252	70.7	44.8
SNX9	0.252	66.4	50.8
DBI	0.252	78.2	59.8
CYP1B1	0.252	87	93.5
PROCR	0.252	34.7	12.4
SAMD11	0.252	37.6	14.8
WISP2	0.252	22.8	11.1
TPGS2	0.251	49.6	21.1
CAPZB	0.251	88.8	73.6
RTKN2	0.251	16.1	2.9
TNFRSF12A	0.250	70.8	60.9
PRELID1	0.250	77.1	53.9
TMEM14B	0.250	57.1	27.1
PRKCSH	-0.250	39.3	62
FMOD	-0.251	9.4	20.6
YBX3	-0.251	71.8	86.6
PMAIP1	-0.251	4	20.6
MT-CO1	-0.253	99.7	99.6
IFITM3	-0.254	85.8	94.3
AHR	-0.255	17.4	31.5
DDX3Y	-0.258	0.4	20.4
ASPH	-0.260	41.4	60.2
FAM20C	-0.261	18.8	31.8
GGT5	-0.262	3.3	20.4
N4BP2L2	-0.263	56.3	77.5
HLA-A	-0.264	91	96.7
NAP1L1	-0.264	80.8	91.1
RPLP0	-0.267	99.9	100
MT-CO2	-0.270	99.6	99.6
LAMB2	-0.270	61.2	82
HSP90B1	-0.273	86	95.8
DDR2	-0.273	51.7	77.5
DST	-0.275	68.6	87.8
DTWD1	-0.276	33.3	49.3
IL6ST	-0.279	42.3	66.7
GABPB1-AS1	-0.279	15.4	30.4
CHI3L2	-0.280	1.5	13.1

COL15A1	-0.281	4	19.6
HSPA5	-0.287	73.8	91.8
SPARC	-0.290	95.5	97.9
SLC25A37	-0.293	46.3	68.6
PHLDA1	-0.294	12.4	30.1
RCAN1	-0.295	24	41
P3H2	-0.298	15.5	32.4
COL12A1	-0.300	91.1	97.6
CEBPD	-0.305	41.9	67.4
CA12	-0.314	31.4	54
VMP1	-0.315	40	65.7
EMILIN1	-0.319	38	61.8
PCOLCE	-0.323	84.4	94.9
BGN	-0.352	78.3	94.9
CPXM2	-0.353	1.1	26.6
QSOX1	-0.356	59.7	88
MCL1	-0.363	18.3	42
CPE	-0.364	1.7	18.9
STC1	-0.364	0.7	16.9
COL14A1	-0.368	3.6	25.4
RABGAP1	-0.371	13.6	30.6
ID2	-0.373	58.6	81.8
SERPINH1	-0.374	61.1	87.6
COL5A2	-0.381	57.9	85
NNMT	-0.382	86.6	96.5
TGFBI	-0.384	91.5	97.4
COL3A1	-0.389	72.8	93.8
NAMPT	-0.394	12	32.9
IER3	-0.398	35	70.4
MT-ATP8	-0.401	16.3	37.5
CFH	-0.408	15.7	30.9
RPL23	-0.408	97.8	99.2
JUN	-0.411	30.4	59.4
DUSP1	-0.421	49.3	77.9
RPL37A	-0.422	99.9	100
COL6A2	-0.423	98.3	99.4
NFKBIZ	-0.424	8.5	31.8
RPS11	-0.429	99.5	99.8
RPL27A	-0.431	99.5	99.8
C3	-0.431	3.8	24.3
TM4SF1	-0.439	34.7	72.3
PRELP	-0.440	26.3	51.3
NR4A1	-0.440	10.7	32
PLOD2	-0.441	35.4	68.9
P4HA1	-0.445	24	55.4

WSB1	-0.446	50.2	79.4
ATF4	-0.451	51.2	84.9
RPS2	-0.453	99.9	100
C1R	-0.454	59.8	88.2
RPL31	-0.455	74.7	91.2
NR4A2	-0.458	1.8	27.5
NEAT1	-0.463	99.8	99.6
MT-ND6	-0.468	37	64.4
PDGFRA	-0.474	40.8	76.1
NDUFA4L2	-0.487	70.4	94.3
SOD2	-0.487	32.2	60.9
CCNL1	-0.490	33.4	66.5
RBP4	-0.494	4.8	26.2
SAT1	-0.496	54.7	80.4
SLC38A2	-0.502	54.2	84.8
MT-CYB	-0.505	99.6	99.8
HES1	-0.531	7.1	33.9
CYTL1	-0.544	6.3	28.2
MEST	-0.557	31.4	69.5
PTGS2	-0.565	4.2	27.6
VEGFA	-0.574	18.6	53.4
COL6A1	-0.585	92.8	98.7
TIMP1	-0.590	99.6	99.8
MT-CO3	-0.590	99.5	99.6
EPAS1	-0.594	14.6	56.1
FOSB	-0.607	15.4	48
MT-ATP6	-0.614	99.6	99.8
HAPLN1	-0.624	8.4	38.3
REV3L	-0.629	21.2	56
CYR61	-0.630	49	86.7
MT-ND4	-0.637	99.8	99.9
TNC	-0.644	34.1	74.1
MT-ND3	-0.664	99.9	99.9
FOS	-0.689	50.8	86.2
COL6A3	-0.717	62	93.3
MEG3	-0.723	77	94.7
RPS20	-0.730	62.7	91.6
EGR1	-0.737	23.4	74.3
MT-ND4L	-0.740	48.1	80.8
RPL13A	-0.742	97.4	99.6
SCRG1	-0.764	27.3	73.5
EDIL3	-0.798	1.9	46.8
MTRNR2L12	-0.832	40.4	77.1
CCDC80	-0.837	78.3	95.8
CXCL6	-0.902	10.1	52.3

COMP	-0.933	24.7	67.2
RPS4Y1	-0.951	3.7	62
CPA4	-1.047	6.3	45.1
MT-ND1	-1.165	99.9	99.9
CLU	-1.175	28.2	82.7
MT-ND5	-1.176	79.6	96.9
MT-ND2	-1.271	97.9	99.7
CHI3L1	-1.721	43.4	89.8
