# Supplementary material for: Single-cell analysis reveals heterogeneity of juvenile idiopathic arthritis fibroblast-like synoviocytes with implications for disease subtype
Source: Arthritis Res Ther. 2022 Sep 27;24:225. doi: 10.1186/s13075-022-02913-8 (PMC9513865; doi:10.1186/s13075-022-02913-8)
Supplement: Supplementary file 3 — Additional file 3: Supplemental Table 3. [file 13075_2022_2913_MOESM3_ESM.pdf]

Chondrocyte-like cells
ETB compared to Oligo
*all p-values <0.00009

Gene	Log Fold Change	ETB (% of chondrocyte cells)	Oligo (% of chondrocyte cells)
ABI3BP	0.821	66.70	50.80
FRZB	0.711	25.50	3.40
PIEZO2	0.698	56.60	24.60
TIMP3	0.688	75.80	65.10
CCND1	0.658	67.70	63.00
VCAM1	0.641	64.40	40.90
SULF1	0.618	65.10	49.70
PTGDS	0.596	46.00	16.30
PLEC	0.588	67.20	58.50
P4HB	0.567	72.40	67.30
FBLN1	0.556	57.90	41.60
SH3BGRL3	0.551	89.30	82.30
MEST	0.529	42.70	18.90
COL12A1	0.528	74.80	67.90
IGFBP2	0.514	39.00	16.70
CRIP2	0.512	58.70	41.80
STEAP4	0.500	48.90	26.40
PITX1	0.499	60.90	45.70
MTRNR2L1	0.491	30.90	2.90
COL5A2	0.478	60.40	51.50
ISLR	0.477	67.40	60.30
CPA4	0.461	22.80	4.80
NEK7	0.457	53.10	35.00
CALR	0.441	69.40	63.10
IGFBP5	0.440	69.60	56.30
DKK1	0.438	16.90	8.10
RNASET2	0.428	53.80	42.00
TPM2	0.424	83.60	79.00
PGF	0.421	45.30	33.10
ENO1	0.420	75.20	69.40
MT2A	0.416	82.00	79.10
PKM	0.414	78.60	72.90
KYNU	0.412	41.40	16.90
HSPB1	0.411	73.30	67.00
B4GALT1	0.403	64.40	57.50
MFGE8	0.402	74.10	66.90
EZR	0.401	49.60	25.40
LMO4	0.395	61.20	51.60
GPC1	0.394	56.30	38.50
SERPINE1	0.393	45.50	34.30
CD151	0.391	70.30	63.40
PTGES	0.378	55.90	43.20

CSPG4	0.378	49.90	31.70
NPW	0.373	36.50	18.70
S100A16	0.367	63.50	54.00
CD70	0.364	38.60	18.20
EIF5A	0.364	64.90	56.30
ACTN4	0.360	66.60	59.90
ITGA3	0.353	47.70	22.80
COL5A1	0.352	63.70	56.70
MAP1A	0.346	64.60	55.80
PLXDC2	0.342	52.00	39.10
RCN3	0.342	62.70	51.90
RAPH1	0.338	52.30	39.10
TXN	0.337	82.20	80.70
CD164	0.336	62.80	55.90
CD82	0.334	44.30	21.40
COTL1	0.334	64.20	55.40
COL3A1	0.333	67.70	63.80
GALNT1	0.331	64.00	54.10
CKAP4	0.329	60.70	54.30
MYL9	0.328	75.80	71.60
SERPINH1	0.328	62.00	56.40
HAPLN1	0.327	14.40	5.20
RPL22L1	0.326	56.50	49.60
CD9	0.326	61.20	52.40
PTMS	0.323	72.80	67.80
SERINC2	0.323	47.20	23.00
SPARC	0.322	77.90	70.40
MT1E	0.318	61.40	54.30
BARX1	0.316	42.90	17.20
ROBO1	0.311	46.20	32.70
DSTN	0.311	83.80	81.60
PLOD2	0.309	54.10	41.20
SAMD11	0.308	46.90	27.90
LOXL4	0.307	29.70	12.50
LGALS1	0.307	96.60	94.40
PABPC1	0.299	76.60	73.80
NDNF	0.298	23.60	10.60
ACTB	0.297	91.00	89.10
VKORC1	0.296	70.10	64.10
CRABP2	0.295	36.90	21.20
BSG	0.295	67.40	62.10
CEMIP	0.295	65.90	53.10
ADCY4	0.295	43.80	24.00
CLSTN1	0.293	57.30	47.90
MYH9	0.292	64.10	57.70
TAGLN2	0.289	67.40	59.90
IFITM2	0.289	64.60	59.80

C9ORF16	0.287	60.70	53.20
MAP4	0.284	65.30	58.40
DLX3	0.284	54.30	42.20
PPIB	0.283	68.50	64.10
DPYSL3	0.282	58.40	48.70
LRRC15	0.281	35.80	6.10
PYGB	0.281	54.00	38.10
TUBA1B	0.280	68.00	60.70
NDUFB1	0.280	62.20	55.60
NTNG2	0.279	41.10	15.80
SLC9A3R2	0.279	41.90	24.70
INF2	0.279	55.40	40.00
MYO1C	0.278	60.50	52.00
MAP1B	0.278	68.20	63.80
SEC61A1	0.277	61.90	54.10
FKBP10	0.277	65.30	60.30
CSGALNACT1	0.274	45.90	25.90
MALAT1	0.273	94.30	91.80
KCNQ1OT1	0.273	50.30	39.10
PPIA	0.273	79.00	76.50
C1GALT1	0.272	64.10	58.30
ATP1B1	0.270	46.10	26.90
FAM118A	0.270	51.50	32.70
ADAMTS5	0.268	53.10	41.30
ANKIB1	0.266	53.10	41.20
IL7R	0.265	19.10	4.80
CSF1	0.264	50.50	38.80
WNK1	0.264	61.20	51.90
CLIC4	0.260	65.70	58.70
GAN	0.260	28.70	13.00
CHPF	0.260	61.90	52.80
UAP1	0.259	54.20	44.20
EHD2	0.258	62.70	54.80
TRAM1	0.257	61.50	55.70
DAP	0.256	55.90	45.40
SDC4	0.256	60.40	53.30
MSN	0.252	61.20	53.30
TMSB10	0.252	96.30	96.00
ERRFI1	0.251	54.90	44.00
ATP5ME	0.251	65.40	60.50
FCGRT	-0.251	49.00	54.30
FXYD1	-0.251	12.60	33.40
B2M	-0.252	82.90	84.10
RPL41	-0.253	94.60	97.70
CA12	-0.255	37.80	47.80
LGALS3BP	-0.255	32.20	38.20
HSPB6	-0.256	57.50	58.70

SNHG7	-0.258	44.70	51.50
PLAC9	-0.259	61.40	62.50
CTSL	-0.261	61.70	60.20
SELENOP	-0.262	8.50	26.10
JUNB	-0.264	56.50	58.80
OLFML3	-0.265	25.20	42.30
FBXO32	-0.267	34.30	45.70
NPR3	-0.267	33.50	43.70
EGR1	-0.268	47.40	54.40
FTH1	-0.269	98.10	99.40
PRRX1	-0.270	56.20	58.60
ISCU	-0.270	53.40	58.50
RPL10A	-0.283	78.90	84.60
ADM	-0.285	30.90	40.20
GSN	-0.286	65.00	62.90
ADAMTS1	-0.286	48.50	53.10
RPS26	-0.287	76.30	82.20
SMOC2	-0.293	31.20	38.00
CCDC85B	-0.295	66.60	69.50
AKR1C3	-0.296	19.70	39.80
CD99	-0.298	71.00	72.20
SRPX	-0.300	15.40	35.10
BASP1	-0.303	50.40	54.50
SCUBE3	-0.305	16.70	28.60
ANGPTL2	-0.306	32.80	44.00
AKR1C2	-0.309	29.70	37.10
LGALS3	-0.309	69.90	74.50
CD44	-0.311	66.50	67.70
CYBRD1	-0.315	59.20	60.10
PLPP1	-0.323	39.10	45.80
ZFP36	-0.323	42.20	48.60
DUSP1	-0.325	44.90	52.40
FBN1	-0.326	64.50	63.40
RBMS1	-0.327	57.20	59.50
MT-CYB	-0.339	98.30	98.50
COL14A1	-0.345	14.90	33.40
SERPINE2	-0.352	70.70	76.30
TGFBI	-0.358	68.20	72.00
SOD2	-0.371	42.20	50.30
ENPP2	-0.372	32.80	45.60
FTL	-0.373	96.50	99.10
BTG1	-0.375	49.60	56.50
CXCL12	-0.383	37.10	52.40
SERPING1	-0.387	32.60	40.90
AEBP1	-0.393	26.80	46.00
CEBPD	-0.395	42.40	53.20
CTSK	-0.400	16.60	42.20

CFD	-0.401	9.70	22.70
PCOLCE2	-0.405	45.40	51.40
CYR61	-0.408	41.40	51.80
MMP2	-0.420	71.90	74.30
C1S	-0.429	59.60	59.50
SNX9	-0.451	56.80	61.40
MT-ND5	-0.471	92.90	91.20
PLA2G2A	-0.472	1.00	10.50
C1R	-0.476	60.50	60.20
ZFP36L1	-0.486	53.10	59.50
AKR1C1	-0.493	33.40	48.70
NR4A1	-0.496	23.80	34.10
WISP2	-0.501	14.60	37.20
PTX3	-0.511	37.80	51.50
FGF7	-0.513	33.40	50.60
SOD3	-0.516	18.20	41.30
POSTN	-0.589	42.40	61.50
GREM2	-0.623	11.50	45.80
EFEMP1	-0.673	56.00	60.90
DCN	-0.674	73.40	77.60
GREM1	-0.683	34.80	67.30
AKAP12	-0.685	51.40	60.50
PLPP3	-0.800	49.60	59.20
CRLF1	-0.814	54.10	63.10
MFAP5	-0.845	41.30	58.70
TRH	-0.897	3.00	14.60
RPS4Y1	-1.333	1.30	70.20
CHI3L1	-1.443	36.10	57.20
