# Supplementary material for: Single-cell analysis reveals heterogeneity of juvenile idiopathic arthritis fibroblast-like synoviocytes with implications for disease subtype
Source: Arthritis Res Ther. 2022 Sep 27;24:225. doi: 10.1186/s13075-022-02913-8 (PMC9513865; doi:10.1186/s13075-022-02913-8)
Supplement: Supplementary file 4 — Additional file 4: Supplemental Table 4. [file 13075_2022_2913_MOESM4_ESM.pdf]

Chondrocyte-like cells
ETB compared to Poly
*all p-values <0.0004

Gene	Log Fold Change	ETB (% of chondrocyte cells)	Poly (% of chondrocyte cells)
S100A4	1.245	89.60	39.00
TIMP3	1.237	75.80	48.20
NBL1	1.122	72.40	40.70
ABI3BP	1.082	66.70	38.90
XIST	1.006	63.70	12.80
STEAP4	0.970	48.90	6.50
CCND1	0.869	67.70	42.60
CD248	0.812	61.10	30.70
PTGDS	0.808	46.00	8.90
PIEZO2	0.795	56.60	20.60
ADIRF	0.782	79.50	60.50
IGFBP5	0.756	69.60	47.40
FRZB	0.726	25.50	5.90
SEMA3C	0.715	68.30	47.80
PGF	0.709	45.30	16.40
VCAM1	0.684	64.40	35.70
TNXB	0.682	49.00	11.60
GALNT1	0.681	64.00	40.30
SH3BGRL3	0.647	89.30	81.40
PLEC	0.643	67.20	53.20
PRG4	0.641	27.90	4.20
PTGES	0.639	55.90	17.20
MYL9	0.632	75.80	57.50
HSPB1	0.631	73.30	54.60
IGFBP6	0.625	77.30	66.90
PENK	0.624	57.00	26.60
LTBP2	0.604	61.00	34.50
ITGBL1	0.589	60.10	29.10
MTRNR2L12	0.583	90.50	82.60
MAP1A	0.582	64.60	38.40
AHNAK	0.580	71.40	62.30
MFGE8	0.568	74.10	66.20
CLEC3B	0.566	51.30	11.90
RNASET2	0.549	53.80	21.80
ECM1	0.537	59.50	26.70
FAM118A	0.526	51.50	10.50
CD9	0.525	61.20	46.20
DLX3	0.524	54.30	11.30
KYNU	0.522	41.40	5.80
MTRNR2L1	0.518	30.90	1.10
B4GALT1	0.516	64.40	51.10
POLR2L	0.513	79.20	67.70

CD164	0.499	62.80	47.00
HEG1	0.493	58.60	26.60
MT-ATP8	0.492	74.80	49.30
FBLN1	0.491	57.90	46.90
LOX	0.491	73.00	61.60
IFITM2	0.490	64.60	46.60
S100A13	0.488	74.80	57.50
CREB5	0.485	52.30	21.40
NEK7	0.479	53.10	21.30
PLXDC2	0.478	52.00	22.00
GPC1	0.472	56.30	25.70
MXRA5	0.471	43.10	9.80
MT2A	0.469	82.00	78.40
GPX4	0.466	78.00	61.60
LTBP3	0.462	63.00	48.10
ACTN4	0.462	66.60	51.70
FST	0.461	55.90	34.50
ITGB5	0.461	61.50	36.00
ENG	0.458	62.60	44.60
CRABP2	0.458	36.90	5.00
FN1	0.457	91.80	90.10
MT-ND6	0.457	83.80	63.00
ANKH	0.455	57.00	27.40
EPB41L1	0.454	55.40	17.50
SULF1	0.452	65.10	53.30
ISLR	0.449	67.40	59.30
ACTB	0.445	91.00	89.70
GSN	0.443	65.00	46.80
PKM	0.443	78.60	72.10
PTMS	0.438	72.80	59.40
CRLF1	0.433	54.10	29.20
IGFBP2	0.432	39.00	19.10
SDC4	0.429	60.40	44.10
CAVIN1	0.429	75.70	67.10
FSTL1	0.426	76.10	73.20
DPYSL3	0.425	58.40	29.30
SEMA5A	0.419	44.60	7.60
S100A16	0.419	63.50	39.20
NDUFB1	0.416	62.20	41.50
CSF1	0.409	50.50	18.80
CD151	0.408	70.30	62.10
VKORC1	0.408	70.10	60.60
ITGB8	0.408	41.90	7.90
CAV1	0.408	71.90	51.50
CD70	0.406	38.60	9.90
TMA7	0.404	72.20	60.30
DSTN	0.404	83.80	79.10

THBS2	0.403	43.40	19.40
EZR	0.402	49.60	16.30
TXN	0.401	82.20	77.70
COL1A2	0.399	87.70	85.80
MT-ND4L	0.399	90.10	82.70
CRIP2	0.398	58.70	36.60
CAPZB	0.398	67.30	50.70
SERINC2	0.397	47.20	10.20
ADCY4	0.397	43.80	11.30
CAPNS1	0.395	65.60	47.20
ARPC1B	0.395	63.10	39.40
COX7A1	0.394	58.40	33.30
NPW	0.394	36.50	14.60
SEMA3B	0.394	48.50	16.60
SPTAN1	0.393	61.40	37.50
UBL5	0.392	71.10	58.40
OAF	0.391	49.60	16.30
SELENON	0.391	56.20	24.10
CKB	0.391	59.70	33.60
SERPINE1	0.386	45.50	25.80
ANPEP	0.385	57.10	30.30
HSPB6	0.384	57.50	34.20
CAPG	0.384	63.00	40.70
CALM1	0.383	67.20	49.90
ANKIB1	0.382	53.10	25.20
SLC7A8	0.382	45.20	17.00
BZW1	0.380	67.20	56.20
PDE1A	0.380	45.30	9.00
EFHD2	0.380	56.60	26.60
NDNF	0.375	23.60	3.80
CLIC3	0.374	51.50	25.50
CHPF	0.373	61.90	40.40
TMEM50A	0.372	63.80	48.30
MMP2	0.371	71.90	64.50
COL12A1	0.370	74.80	74.10
LMNA	0.370	75.70	71.20
SIPA1L1	0.368	59.20	31.80
PPDPF	0.367	78.40	70.50
MYH9	0.366	64.10	48.20
ATP5ME	0.366	65.40	49.80
GPX3	0.364	43.80	13.60
VAT1	0.363	62.90	39.40
RAPH1	0.362	52.30	26.20
MFAP5	0.362	41.30	19.70
ACKR3	0.362	44.10	17.70
MINOS1	0.359	64.90	46.80
PITX1	0.359	60.90	43.60

ACAN	0.357	56.60	51.30
GUK1	0.357	74.40	58.40
SERF2	0.356	92.70	91.50
EIF5A	0.356	64.90	44.90
TIMP2	0.356	72.30	64.50
AHNAK2	0.355	53.10	21.50
SERPINE2	0.355	70.70	66.40
HTRA1	0.352	67.70	59.10
CAPN2	0.349	59.70	35.70
NDUFA3	0.349	59.70	34.90
CST3	0.348	70.50	64.20
GPR153	0.347	42.90	4.70
NDUFS5	0.346	70.40	56.50
SSC5D	0.344	54.80	24.30
CERCAM	0.344	62.30	44.60
MYL12B	0.343	75.10	63.70
BSG	0.340	67.40	61.10
MYO1C	0.339	60.50	39.60
CSGALNACT1	0.339	45.90	12.10
CD82	0.338	44.30	13.60
S100A11	0.337	88.60	86.10
KANK2	0.337	58.60	29.20
TAGLN2	0.337	67.40	49.50
SELENOW	0.337	64.80	42.20
DLGAP4	0.337	59.20	33.90
LOXL4	0.333	29.70	6.30
MT-ND3	0.332	95.60	94.40
JCAD	0.331	46.90	13.40
AK1	0.330	51.50	17.40
MRC2	0.329	63.60	57.30
SAMD11	0.329	46.90	18.80
WNT5A	0.322	55.70	34.60
UGDH	0.322	57.10	33.30
BARX1	0.319	42.90	15.80
SPTBN1	0.316	60.90	41.60
CMKLR1	0.315	42.80	10.60
ROBO1	0.314	46.20	19.10
OGN	0.311	37.80	12.60
MSN	0.311	61.20	39.60
PHF20	0.311	55.30	26.90
ROMO1	0.310	61.00	39.00
LIMS2	0.310	46.60	10.40
CUX1	0.309	58.40	33.90
CSPG4	0.308	49.90	23.50
PPIA	0.307	79.00	74.10
MAP1B	0.307	68.20	54.30
LRRC15	0.306	35.80	1.30

EHD1	0.306	49.00	19.40
UBA1	0.305	56.40	27.70
LMO7	0.305	66.20	46.70
ATOX1	0.304	64.10	44.00
COPZ2	0.303	65.80	46.00
INF2	0.302	55.40	25.40
MAPK3	0.302	55.40	25.80
FLNB	0.301	55.00	31.90
PALLD	0.301	57.80	30.20
CRIM1	0.301	63.20	51.00
NDUFA13	0.300	62.00	46.40
C1GALT1	0.300	64.10	54.40
ARHGAP1	0.299	54.20	24.40
SAMD9	0.299	45.60	11.60
EPB41L2	0.299	56.70	34.90
CEMIP	0.298	65.90	45.00
BAG2	0.298	54.10	27.00
ADAMTSL1	0.298	53.50	22.40
LIMA1	0.297	66.90	53.70
NFIX	0.296	66.30	50.90
LOXL1	0.296	61.50	48.10
FNDC1	0.295	39.20	17.70
COX6C	0.295	66.60	55.20
HSD3B7	0.295	40.90	8.80
ELOB	0.294	72.40	63.90
LRP1	0.291	66.00	60.60
NDUFA1	0.291	63.20	48.30
CAVIN3	0.289	65.20	51.80
ATP1B1	0.289	46.10	22.30
MXRA8	0.286	67.30	57.60
RAB23	0.286	48.80	16.50
PIEZO1	0.286	55.00	26.90
PLAU	0.285	40.20	16.40
WNK1	0.285	61.20	41.40
DDAH2	0.285	61.20	34.80
YWHAE	0.285	66.80	54.30
ANXA5	0.284	78.30	72.40
SMOC2	0.282	31.20	7.40
GLIS2	0.280	45.60	13.80
ARHGDIA	0.279	64.90	50.40
PTGIS	0.278	34.40	10.10
STAC2	0.278	32.70	3.10
ITGA3	0.277	47.70	20.30
THBS4	0.277	18.30	1.30
CLSTN1	0.276	57.30	37.30
CFL1	0.276	76.40	67.50
UCHL1	0.276	39.90	21.50

PYCARD	0.275	45.20	22.90
UQCRQ	0.275	67.60	54.50
ITPR3	0.274	47.40	17.20
FBLN2	0.274	54.10	31.40
PET100	0.272	57.00	31.30
UQCR11	0.272	64.30	50.70
PRUNE2	0.272	52.20	26.60
SHISA5	0.271	57.60	35.70
HERC4	0.271	57.80	38.50
GPNMB	0.270	55.20	30.20
SLIRP	0.270	57.80	36.00
NFE2L1	0.270	59.80	42.50
LRRN4CL	0.270	44.00	11.70
CIT	0.269	33.00	4.90
ACTR1A	0.268	53.70	23.30
FGFR1	0.264	61.10	46.70
HSPA1A	0.264	51.30	25.90
PRIM2	0.264	41.90	13.50
VEGFC	0.263	54.10	30.80
HAS3	0.263	18.30	1.90
C9ORF16	0.263	60.70	40.80
G6PD	0.262	53.70	25.50
ZFP36L2	0.262	59.40	42.20
THY1	0.262	64.10	52.10
MAP4	0.262	65.30	50.60
CD276	0.261	53.40	28.20
APLP2	0.261	65.50	59.20
SKIL	0.261	50.10	20.50
VSIR	0.260	48.70	23.10
CLTB	0.260	56.20	32.70
UAP1	0.259	54.20	33.30
AKAP12	0.259	51.40	30.60
CRIP1	0.258	34.00	3.20
CLIC4	0.257	65.70	52.20
DLC1	0.256	57.70	35.40
MTCH1	0.255	63.20	43.10
SMPD1	0.254	54.50	31.40
PRDX1	0.252	70.80	62.40
NFATC4	0.252	55.70	34.70
PEBP1	0.251	67.60	50.40
TUBA1B	0.250	68.00	53.50
ANXA2	0.250	85.00	82.80
PDLIM7	0.250	60.40	35.70
ABL2	-0.255	41.90	40.80
LAMB1	-0.255	41.60	41.30
SGCE	-0.256	38.40	40.20
SMOC1	-0.256	22.90	28.60

PNRC1	-0.258	50.50	47.30
TSPYL2	-0.260	24.30	27.30
TWIST1	-0.260	42.20	41.30
ASPH	-0.260	53.90	48.90
RPL3	-0.261	90.10	93.60
COL1A1	-0.263	80.30	85.00
VMP1	-0.263	56.70	52.00
NFIL3	-0.265	16.80	28.50
RHOBTB3	-0.265	44.70	42.40
PGK1	-0.267	52.10	51.00
NPM1	-0.267	74.30	76.40
RPL15	-0.267	87.80	92.60
RPS3A	-0.268	86.40	90.90
RPS7	-0.271	81.80	85.60
RPL10	-0.271	94.40	96.80
SLC16A3	-0.273	48.70	45.10
FGFRL1	-0.275	38.80	38.90
UGCG	-0.277	38.80	39.00
FOXQ1	-0.277	11.30	22.10
COL6A3	-0.279	66.10	67.30
SLPI	-0.280	4.50	13.60
TPI1	-0.282	67.50	68.10
ALCAM	-0.283	33.30	36.80
ADH5	-0.285	54.40	49.80
RPS9	-0.285	83.90	89.10
CP	-0.286	1.40	12.00
AEBP1	-0.286	26.80	34.70
ZNF395	-0.287	23.80	31.50
TIPARP	-0.287	15.50	26.60
RPS2	-0.289	90.80	93.00
GJA1	-0.289	46.10	46.80
TRPS1	-0.289	46.20	43.90
PTGFR	-0.290	9.90	23.80
PLIN2	-0.291	44.40	43.30
PDIA5	-0.292	45.80	44.20
C1S	-0.292	59.60	56.80
SIX1	-0.294	33.20	39.80
MCL1	-0.295	50.70	46.40
MT-ND1	-0.296	95.00	94.80
HMOX1	-0.300	41.40	41.60
RPS5	-0.300	80.30	84.40
RPS12	-0.300	94.00	97.00
COL6A2	-0.301	71.10	76.30
FAM20C	-0.304	44.10	41.10
JUN	-0.304	44.70	48.70
RPL23A	-0.304	78.80	84.60
RPL31	-0.304	66.00	65.90

FTH1	-0.305	98.10	99.60
DTWD1	-0.308	51.40	47.70
GLRX	-0.309	47.10	46.20
NFASC	-0.310	6.10	25.50
EEF1A1	-0.311	98.90	99.50
RPL21	-0.313	79.70	83.90
DCN	-0.315	73.40	79.10
NNMT	-0.315	70.50	75.30
LOXL2	-0.315	42.40	42.40
SLC38A1	-0.316	10.30	30.00
ID4	-0.319	17.30	29.00
BASP1	-0.321	50.40	49.00
STEAP1	-0.327	4.40	25.30
MDK	-0.331	24.70	34.50
CACNA1A	-0.334	11.50	28.50
SCRG1	-0.334	54.60	52.60
WSB1	-0.336	61.00	58.30
BNIP3L	-0.337	52.60	51.60
COL11A1	-0.337	5.60	21.90
ADM	-0.340	30.90	40.60
EIF1	-0.341	81.50	87.10
COL6A1	-0.342	69.90	73.10
CD44	-0.343	66.50	68.20
HSPA5	-0.344	61.00	60.90
IL6	-0.346	5.50	16.50
PMAIP1	-0.348	7.10	25.40
CXCL3	-0.349	1.00	14.10
SOX9	-0.350	4.00	19.70
GABPB1-AS1	-0.353	36.30	39.80
TM4SF1	-0.358	47.40	50.40
GGT5	-0.361	6.30	25.50
TNC	-0.361	55.00	53.00
SLC38A2	-0.363	60.90	59.20
BNIP3	-0.366	48.60	51.00
MEG3	-0.366	68.80	72.30
RPL10A	-0.372	78.90	83.30
DDX3Y	-0.376	0.00	26.50
NDRG1	-0.380	39.60	43.80
RCAN1	-0.381	38.10	43.10
COL15A1	-0.381	4.00	23.20
SLC7A5	-0.381	21.60	34.80
HIF1A	-0.381	55.60	57.60
PLOD2	-0.381	54.10	50.90
ADAMTS6	-0.382	21.10	29.40
COL14A1	-0.389	14.90	31.00
ENPP2	-0.390	32.80	40.50
C1R	-0.390	60.50	59.80

FTL	-0.393	96.50	99.00
STC2	-0.395	35.60	44.50
BTG1	-0.401	49.60	50.80
P3H2	-0.410	28.00	38.10
PTX3	-0.410	37.80	47.30
PHLDA1	-0.410	25.30	36.00
CHI3L2	-0.414	2.00	17.00
RPS20	-0.417	65.70	68.90
ELL2	-0.421	41.50	49.50
STK17B	-0.425	21.90	36.00
RABGAP1	-0.427	36.80	38.30
IL13RA2	-0.427	21.50	33.50
RPL13A	-0.428	78.90	84.10
RPL7	-0.436	72.20	78.30
P4HA1	-0.439	46.50	47.30
FMOD	-0.449	10.70	26.70
CPXM2	-0.451	8.70	33.50
MEST	-0.457	42.70	48.80
CCL2	-0.462	19.90	31.00
BGN	-0.466	62.70	68.30
NFKBIZ	-0.466	30.50	40.10
FOSB	-0.473	36.80	47.00
SOD3	-0.474	18.20	30.20
CA12	-0.477	37.80	46.80
PDGFRA	-0.478	53.10	55.40
FOS	-0.479	51.80	61.50
STC1	-0.498	0.60	20.90
PLAGL1	-0.506	48.00	49.20
ID2	-0.512	54.30	57.40
CFH	-0.512	40.00	41.20
RBP4	-0.513	5.40	32.00
CPE	-0.518	1.50	24.00
PAPPA	-0.521	5.00	27.40
CEBPD	-0.526	42.40	50.00
PLPP3	-0.542	49.60	56.40
ADAMTS1	-0.543	48.50	56.90
GREM1	-0.545	34.80	56.40
TIMP1	-0.547	79.10	86.10
EGR1	-0.549	47.40	55.70
HAPLN1	-0.554	14.40	40.40
EPAS1	-0.555	41.80	48.90
CCDC80	-0.578	68.90	75.10
NAMPT	-0.580	28.70	42.40
NR4A1	-0.585	23.80	38.30
CCNL1	-0.617	50.10	53.00
DUSP1	-0.617	44.90	55.30
C3	-0.648	1.10	29.50

SOD2	-0.650	42.20	51.30
NR4A2	-0.653	3.80	33.40
MMP3	-0.673	1.30	14.60
HES1	-0.680	18.30	40.70
TNFAIP6	-0.704	45.40	53.80
REV3L	-0.721	42.80	49.90
CYTL1	-0.729	11.60	33.40
VEGFA	-0.729	37.20	49.40
CXCL1	-0.772	5.80	31.50
IER3	-0.781	40.70	51.50
CPA4	-0.798	22.80	39.80
EFEMP1	-0.848	56.00	67.60
PTGS2	-0.858	2.10	33.20
CLU	-0.898	50.90	59.80
COMP	-0.917	40.40	51.30
CYR61	-0.919	41.40	61.00
EDIL3	-0.936	11.50	45.10
NDUFA4L2	-0.951	61.50	79.90
TGFBI	-0.984	68.20	79.80
RPS4Y1	-1.087	1.30	48.00
CXCL6	-1.316	5.30	43.60
CHI3L1	-2.427	36.10	76.50
