# Supplementary material for: Single-cell analysis reveals heterogeneity of juvenile idiopathic arthritis fibroblast-like synoviocytes with implications for disease subtype
Source: Arthritis Res Ther. 2022 Sep 27;24:225. doi: 10.1186/s13075-022-02913-8 (PMC9513865; doi:10.1186/s13075-022-02913-8)
Supplement: Supplementary file 5 — Additional file 5: Supplemental Table 5. [file 13075_2022_2913_MOESM5_ESM.pdf]

Chondrocyte-like cells
Oligo compared to Poly
*all p-values <0.0021

Gene	Log Fold Change	Oligo (% of chondrocyte cells)	Poly (% of chondrocyte cells)
CRLF1	1.247	63.10	29.20
MFAP5	1.207	58.70	19.70
S100A4	1.013	86.90	39.00
POSTN	0.947	61.50	49.10
AKAP12	0.944	60.50	30.60
NBL1	0.940	70.30	40.70
TRH	0.908	14.60	0.50
MMP2	0.791	74.30	64.50
TNXB	0.761	44.60	11.60
CD248	0.734	53.40	30.70
GSN	0.729	62.90	46.80
IGFBP6	0.708	74.30	66.90
SERPINE2	0.707	76.30	66.40
MTRNR2L12	0.699	88.30	82.60
GREM2	0.642	45.80	8.60
HSPB6	0.640	58.70	34.20
CLEC3B	0.635	43.00	11.90
THBS2	0.590	49.80	19.40
SMOC2	0.575	38.00	7.40
PCOLCE2	0.570	51.40	30.80
ADIRF	0.552	73.50	60.50
TIMP3	0.549	65.10	48.20
SSC5D	0.547	55.80	24.30
HTRA1	0.536	66.20	59.10
ITGBL1	0.519	53.50	29.10
SEMA3C	0.517	61.70	47.80
PRG4	0.501	19.40	4.20
PTGIS	0.498	37.20	10.10
COX7A1	0.482	60.90	33.30
GPX4	0.480	79.60	61.60
MT-ND3	0.472	94.30	94.40
LRRN4CL	0.471	48.40	11.70
STEAP4	0.470	26.40	6.50
WNT5A	0.465	49.20	34.60
SNX9	0.457	61.40	43.80
ECM1	0.448	54.10	26.70
PODN	0.439	44.10	10.80
HEG1	0.429	52.90	26.60
S100A13	0.428	73.00	57.50
GPNMB	0.424	54.80	30.20
MT-ND4L	0.420	88.20	82.70
CD81	0.414	70.90	67.80

CFD	0.414	22.70	3.90
PENK	0.412	44.80	26.60
ANKH	0.407	50.70	27.40
COL1A2	0.406	87.50	85.80
ZFP36L1	0.399	59.50	45.70
WISP2	0.397	37.20	14.30
PDGFRB	0.395	51.90	26.50
MT-ND6	0.394	81.90	63.00
POLR2L	0.392	79.00	67.70
KANK2	0.391	56.30	29.20
TIMP2	0.390	69.10	64.50
ITGB5	0.378	57.60	36.00
LTBP2	0.370	53.20	34.50
FGF7	0.369	50.60	29.00
AHNAK	0.367	68.90	62.30
C16ORF45	0.366	51.20	21.20
MINOS1	0.363	63.20	46.80
DCN	0.360	77.60	79.10
VGLL3	0.352	47.60	14.40
CXCL12	0.352	52.40	27.60
AHNAK2	0.351	47.00	21.50
GALNT1	0.351	54.10	40.30
VSIR	0.350	43.50	23.10
SERPING1	0.347	40.90	22.30
PLA2G2A	0.346	10.50	4.90
ANGPTL2	0.345	44.00	18.90
HSPB7	0.342	49.40	38.30
CREB5	0.341	42.80	21.40
NFIX	0.339	63.20	50.90
NPR3	0.336	43.70	18.50
FN1	0.330	88.50	90.10
MEDAG	0.329	42.00	13.30
VEGFB	0.328	58.40	36.20
MXRA8	0.326	63.20	57.60
RBPJ	0.322	64.80	58.10
VCAN	0.321	57.70	45.60
ENG	0.321	57.00	44.60
VEGFC	0.321	54.60	30.80
SH3D19	0.320	47.40	20.30
FST	0.319	53.10	34.50
ADD3	0.319	51.80	27.50
IGFBP5	0.316	56.30	47.40
AKR1C3	0.313	39.80	13.20
RAB31	0.313	49.10	20.70
SPTBN1	0.310	57.50	41.60
CST3	0.307	67.50	64.20
ARPC1B	0.305	58.90	39.40

MYL9	0.303	71.60	57.50
ACKR3	0.303	36.70	17.70
NDUFS5	0.299	69.70	56.50
LGALS3BP	0.299	38.20	18.70
CTSB	0.299	66.30	60.70
LRP1	0.294	61.80	60.60
CAPG	0.294	59.10	40.70
DDAH2	0.293	57.10	34.80
LTBP3	0.293	58.50	48.10
HTRA3	0.292	27.70	6.40
PLAU	0.291	38.90	16.40
CD99	0.289	72.20	67.70
PGF	0.288	33.10	16.40
OLFML2B	0.288	37.90	10.10
EPB41L1	0.284	44.80	17.50
CAPZB	0.284	64.70	50.70
MARCKS	0.284	64.80	55.90
ARL2	0.281	57.60	37.60
PRRX1	0.281	58.60	43.80
CTSK	0.281	42.20	18.30
CYBRD1	0.281	60.10	52.50
C12ORF75	0.280	58.80	35.60
ATP5F1E	0.279	78.00	69.30
CAV1	0.277	67.90	51.50
ITGB8	0.277	32.00	7.90
LGALS3	0.276	74.50	67.00
FBLN2	0.275	48.80	31.40
RGS3	0.273	50.30	31.40
CBR3	0.271	40.70	14.40
KCTD12	0.271	29.70	5.80
ANXA1	0.269	70.00	62.60
ITM2B	0.268	64.10	61.70
MXRA5	0.265	31.30	9.80
CKB	0.265	51.30	33.60
LOX	0.263	65.80	61.60
MYO1E	0.261	47.20	19.90
ABI3BP	0.261	50.80	38.90
PTGES	0.260	43.20	17.20
OAF	0.260	41.80	16.30
RPS10	0.260	72.80	60.60
S100A10	0.259	89.50	89.00
MT-ATP8	0.259	71.50	49.30
SPTAN1	0.258	55.50	37.50
PLPP3	0.258	59.20	56.40
MT-ND5	0.258	91.20	91.40
KAZALD1	0.257	44.30	20.00
FAM118A	0.256	32.70	10.50

LAPTM4A	0.255	61.40	53.90
THBS4	0.254	11.80	1.30
ANXA4	0.252	50.70	29.30
ACAN	0.252	57.10	51.30
SMURF2	0.252	43.20	22.40
TGFBR3	0.250	36.50	10.70
IL6	-0.251	8.70	16.50
SLC3A2	-0.253	48.30	47.80
PMAIP1	-0.253	13.90	25.40
TBL1XR1	-0.256	46.80	45.40
ZNF292	-0.257	40.60	39.80
RPL37A	-0.257	87.00	87.60
ADAMTS1	-0.258	53.10	56.90
SLC7A11	-0.259	13.30	23.50
TNC	-0.259	49.20	53.00
STK17B	-0.262	29.30	36.00
HIF1A	-0.263	56.10	57.60
SLPI	-0.265	5.30	13.60
IL6ST	-0.266	52.70	51.30
PGK1	-0.266	52.50	51.00
RPS11	-0.266	78.50	80.40
RHOBTB3	-0.267	40.80	42.40
VMP1	-0.269	52.60	52.00
DTWD1	-0.269	50.80	47.70
PLOD1	-0.277	44.20	44.20
JUN	-0.277	46.20	48.70
SOD2	-0.279	50.30	51.30
EGR1	-0.281	54.40	55.70
ENO1	-0.286	69.40	74.30
FBN2	-0.288	22.90	32.50
STC2	-0.289	41.80	44.50
GARS	-0.291	47.40	46.80
DUSP1	-0.292	52.40	55.30
CXCL3	-0.297	3.20	14.10
TPI1	-0.299	67.20	68.10
FKBP10	-0.300	60.30	62.10
GABPB1-AS1	-0.302	37.80	39.80
ADAMTS5	-0.303	41.30	42.60
MTHFD2	-0.307	25.80	34.90
RCN3	-0.313	51.90	54.30
COL11A1	-0.316	10.50	21.90
RPL23	-0.318	73.10	75.40
COL1A1	-0.318	80.40	85.00
LMAN1	-0.318	54.50	56.10
COL6A1	-0.326	68.80	73.10
FOS	-0.328	54.00	61.50
P4HB	-0.329	67.30	71.50

QSOX1	-0.337	57.30	60.70
SOX9	-0.338	4.70	19.70
SAT1	-0.343	59.60	59.00
ID2	-0.345	55.70	57.40
ATF4	-0.350	59.90	61.50
KCNQ1OT1	-0.351	39.10	42.40
GGT5	-0.353	7.40	25.50
WSB1	-0.353	57.40	58.30
SLC38A1	-0.359	9.90	30.00
RPL31	-0.362	63.80	65.90
CHI3L2	-0.365	7.70	17.00
CDC42EP3	-0.367	42.80	50.30
CFH	-0.369	40.20	41.20
PPIB	-0.369	64.10	68.40
EIF4EBP1	-0.372	33.10	40.80
PAPPA	-0.378	18.20	27.40
RPL27A	-0.380	80.30	83.90
RPS2	-0.381	92.60	93.00
RABGAP1	-0.385	37.20	38.30
TPM2	-0.386	79.00	83.30
SLC16A3	-0.387	38.40	45.10
XIST	-0.391	0.40	12.80
SPARC	-0.398	70.40	77.90
COL3A1	-0.400	63.80	71.40
BNIP3	-0.400	46.60	51.00
GAPDH	-0.400	94.40	97.10
SLC7A5	-0.400	18.80	34.80
TM4SF1	-0.402	44.50	50.40
NFKBIZ	-0.407	30.20	40.10
IGFBP3	-0.408	87.60	92.50
MALAT1	-0.412	91.80	94.40
COL15A1	-0.412	2.90	23.20
NEAT1	-0.423	83.40	88.40
MEG3	-0.427	64.90	72.30
RCAN1	-0.434	33.80	43.10
CPXM2	-0.435	10.30	33.50
IL13RA2	-0.442	15.00	33.50
HSP90B1	-0.444	62.30	67.70
RBP4	-0.446	14.30	32.00
NAMPT	-0.447	30.90	42.40
TIMP1	-0.448	80.00	86.10
RPL13A	-0.450	80.90	84.10
ELL2	-0.454	39.80	49.50
CLU	-0.459	49.90	59.80
SERPINH1	-0.461	56.40	59.40
P3H2	-0.469	21.50	38.10
STC1	-0.472	2.10	20.90

PLAGL1	-0.476	48.90	49.20
MTRNR2L8	-0.478	71.10	55.00
CCNL1	-0.484	50.50	53.00
SCRG1	-0.493	40.10	52.60
HSPA5	-0.501	56.60	60.90
NR4A2	-0.501	15.50	33.40
CYR61	-0.511	51.80	61.00
CPE	-0.515	3.80	24.00
FOSB	-0.516	36.50	47.00
BGN	-0.525	56.30	68.30
P4HA1	-0.526	40.10	47.30
CXCL1	-0.529	11.00	31.50
EPAS1	-0.530	36.70	48.90
REV3L	-0.530	46.60	49.90
CCDC80	-0.542	68.70	75.10
TNFAIP6	-0.544	40.50	53.80
RPS20	-0.552	63.40	68.90
VEGFA	-0.581	41.40	49.40
SLC38A2	-0.588	54.20	59.20
HES1	-0.594	19.50	40.70
C3	-0.627	4.90	29.50
TGFBI	-0.627	72.00	79.80
MMP3	-0.664	1.70	14.60
COL5A2	-0.668	51.50	57.40
PLOD2	-0.690	41.20	50.90
IER3	-0.723	35.20	51.50
PTGS2	-0.729	9.40	33.20
COMP	-0.731	42.90	51.30
CYTL1	-0.805	4.50	33.40
NDUFA4L2	-0.873	65.70	79.90
HAPLN1	-0.881	5.20	40.40
EDIL3	-0.888	12.70	45.10
CHI3L1	-0.984	57.20	76.50
MEST	-0.986	18.90	48.80
CXCL6	-1.161	8.70	43.60
CPA4	-1.259	4.80	39.80
